# Metabolic rewiring in MYC-driven medulloblastoma by BET-bromodomain inhibition

**DOI:** 10.1038/s41598-023-27375-z

**Published:** 2023-01-23

**Authors:** Vittoria Graziani, Aida Rodriguez Garcia, Lourdes Sainero Alcolado, Adrien Le Guennec, Marie Arsenian Henriksson, Maria R. Conte

**Affiliations:** 1 Department of Microbiology and Tumor Biology, Biomedicum B7, Karolinska Institutet, 171 65 Stockholm, Sweden; 2 Centre for Biomolecular Spectroscopy, King’s College London, Guy’s Campus, London, SE1 1UL UK; 3 Randall Centre for Cell and Molecular Biophysics, King’s College London, Guy’s Campus, London, SE1 1UL UK; 4 Present Address: Barts Cancer Institute, Queen Mary University of London, John Vane Science Building, Charterhouse Square, London, EC1M 6BQ UK

**Keywords:** Biophysics, Cancer, Cell biology, Diseases, Medical research, Oncology

## Abstract

Medulloblastoma (MB) is the most common malignant brain tumour in children. High-risk MB patients harbouring *MYC* amplification or overexpression exhibit a very poor prognosis. Aberrant activation of MYC markedly reprograms cell metabolism to sustain tumorigenesis, yet how metabolism is dysregulated in MYC-driven MB is not well understood. Growing evidence unveiled the potential of BET-bromodomain inhibitors (BETis) as next generation agents for treating MYC-driven MB, but whether and how BETis may affect tumour cell metabolism to exert their anticancer activities remains unknown. In this study, we explore the metabolic features characterising MYC-driven MB and examine how these are altered by BET-bromodomain inhibition. To this end, we employed an NMR-based metabolomics approach applied to the MYC-driven MB D283 and D458 cell lines before and after the treatment with the BETi OTX-015. We found that OTX-015 triggers a metabolic shift in both cell lines resulting in increased levels of myo-inositol, glycerophosphocholine, UDP-N-acetylglucosamine, glycine, serine, pantothenate and phosphocholine. Moreover, we show that OTX-015 alters ascorbate and aldarate metabolism, inositol phosphate metabolism, phosphatidylinositol signalling system, glycerophospholipid metabolism, ether lipid metabolism, aminoacyl-tRNA biosynthesis, and glycine, serine and threonine metabolism pathways in both cell lines. These insights provide a metabolic characterisation of MYC-driven childhood MB cell lines, which could pave the way for the discovery of novel druggable pathways. Importantly, these findings will also contribute to understand the downstream effects of BETis on MYC-driven MB, potentially aiding the development of new therapeutic strategies to combat medulloblastoma.

## Introduction

Medulloblastoma (MB) is the most common malignant tumour of the central nervous system (CNS) in children, arising from embryonal lesions generated by diverse progenitor cell populations during early brain development^1,2^. It was first described in 1926 as one subset of glioma^3^. Since then, advances in molecular genetics ameliorated our understanding of MB, culminating in a consensus definition of four distinct groups: wingless (WNT), sonic hedgehog (SHH), Group 3 and Group 4, which differ in both molecular and clinical characteristics^4,5^. Unlike the SHH and WNT groups, no common signalling pathway driving the disease has been identified in Group 3 and 4 tumours. The latter ones exhibit the worst prognosis of the four subgroups and recurrent *MYC* amplification or overexpression has been identified as one of the major biomarkers for this high-risk MB patient group with poor clinical outcome^4^. The modest understanding of the molecular mechanisms underpinning tumorigenesis in Group 3 and Group 4 MBs has limited the development of targeted therapies. For moderate and high risk-patients trials includes novel chemotherapeutics (pemetrexed and gemcitabine) after standard chemotherapy and risk-adapted radiotherapy. For recurrent and refractory MB patients two trials combine the use of chemotherapeutics with targeted agents such as prexasertib (NCT04023669), a targeted CHK1/2 inhibitor, and ribociclib (NCT01878617), a cyclin-dependent kinase inhibitor^6^. Current treatments include tumour resection, radiotherapy and chemotherapy. Despite this aggressive multimodal therapy, approximatively 30% of the patients die of disease, while survivors suffer long-term side effects due to the harsh treatments on the developing brain^7,8^.

To date, several preclinical and clinical studies indicated that BET-bromodomain (BRD) inhibitors (BETis) might be considered as next generation agents for treating MYC-driven MB^4,6^. These are small molecules that specifically inhibit proteins in the BRD family which contain two bromodomains capable of recognising acetylated lysine residues in histone tails and recruiting transcriptional factors to promote targeted gene transcription^9^. Their inhibitors prevent the interaction between the bromodomain and the acetyl group, causing the downregulation of certain genes, including *c-MYC*^10^. JQ1 was the first BETi to be developed. This is a cell permeable small molecule that promotes differentiation and halts proliferation in cancer cell lines and in diverse murine tumour models^10–14^. Despite its efficacy, JQ1 has short-lasting effects due to its half-life of around 1 h, greatly limiting the possibilities to translate preclinical findings into clinical benefit^11,14,15^. OTX-015 (MK-8628) is a BETi developed in the past decade that targets BRD 2/3/4^16^. OTX-015 has been synthesised from ( +)-JQ1 by substituting the tert-butyl ester with a para-hydroxyacetamide. This structural change resulted in an improved pharmacokinetic profile^17^ (with an half-life of 6 h)^16,18,19^ which led to its use in several clinical trials including glioblastoma multiforme (GBM), NUT midline carcinoma, triple-negative breast cancer (TNBC), castration-resistant prostate cancer, pancreatic ductal carcinoma and haematological malignancies^20^. OTX-015 causes an early, strong and long lasting reduction of MYC, and active doses were not toxic *in vivo*^16^*.* Furthermore, in GBM xenograft models this compound has been shown to pass through the blood brain barrier and preferentially bind to cancerous tissue, providing a strong pharmacologic basis for OTX-015 use in brain tumour therapy^21^. Moreover, OTX-015 prodrugs with significantly increased antitumor activity and reduced toxicity have recently been developed^22^.

The c-MYC (MYC) transcription factor belongs to the MYC-proto onco-protein family, which also includes MYCN and MYCL^23^. Heterodimerization with MAX is an essential prerequisite for MYC-driven transcription and cellular transformation. The MYC/MAX dimer binds Enhancer box (E-box) sequences regulating the expression levels of genes involved in growth regulatory networks and oncogenic signalling pathways, including energy production and metabolism^23,24^. Specifically, MYC activates genes modulating metabolic processes in order to balance nutrient supply and demand and drive cell fate decisions^25^. This plays a crucial role in tumour development, as cancer cells reprogram their metabolism to fulfil their increased bioenergetic needs, to maintain a high rate of macromolecular biosynthesis, essential for cell growth/division, and to prevent toxic Reactive Oxygen Species (ROS) levels^26^.

To date, despite a plethora of evidence reporting that MYC tunes metabolic networks to foster oncogenesis in diverse tumour types^27^, data remain scarce and limited, and likewise, the metabolic response of BETi treatment of MYC-driven MB remains unexplored. In this work, we investigate changes in metabolism of MYC-driven MB cell lines prior and after treatment with OTX-015. We chose OTX-015 over other inhibitors, *e.g.* JQ1, because of its higher promise in clinical trials. This study contributes to the phenotypical characterisation of MYC-driven MB models and reveals metabolic changes caused by OTX-015, providing new clues of the anticancer mechanisms of action for these compounds.

## Results

### OTX-015 inhibits cell viability and reduces MYC expression in medulloblastoma cell lines

We selected human D283 and D458 MB cell lines as in vitro models of MYC-driven MB. The D283 cell line has been classified as either Group 3 or Group 4 and overexpresses MYC and the transcription factor orthodenticle homeobox 2 (OTX2)^28^. The latter plays a key role in the pathogenesis of anaplastic MB and treatment with all trans-retinoic acid causes OTX2 downregulation and apoptosis^29^. D458 cells belong to Group 3 and are characterised by *MYC* amplification. They derive from the metastasis of a parental tumour and harbour wild type p53^28^ with R72P polymorphism, a common single nucleotide polymorphism (SNP) that results in either an arginine or proline at position 72 of the protein^30,31^.

**Figure 1:**
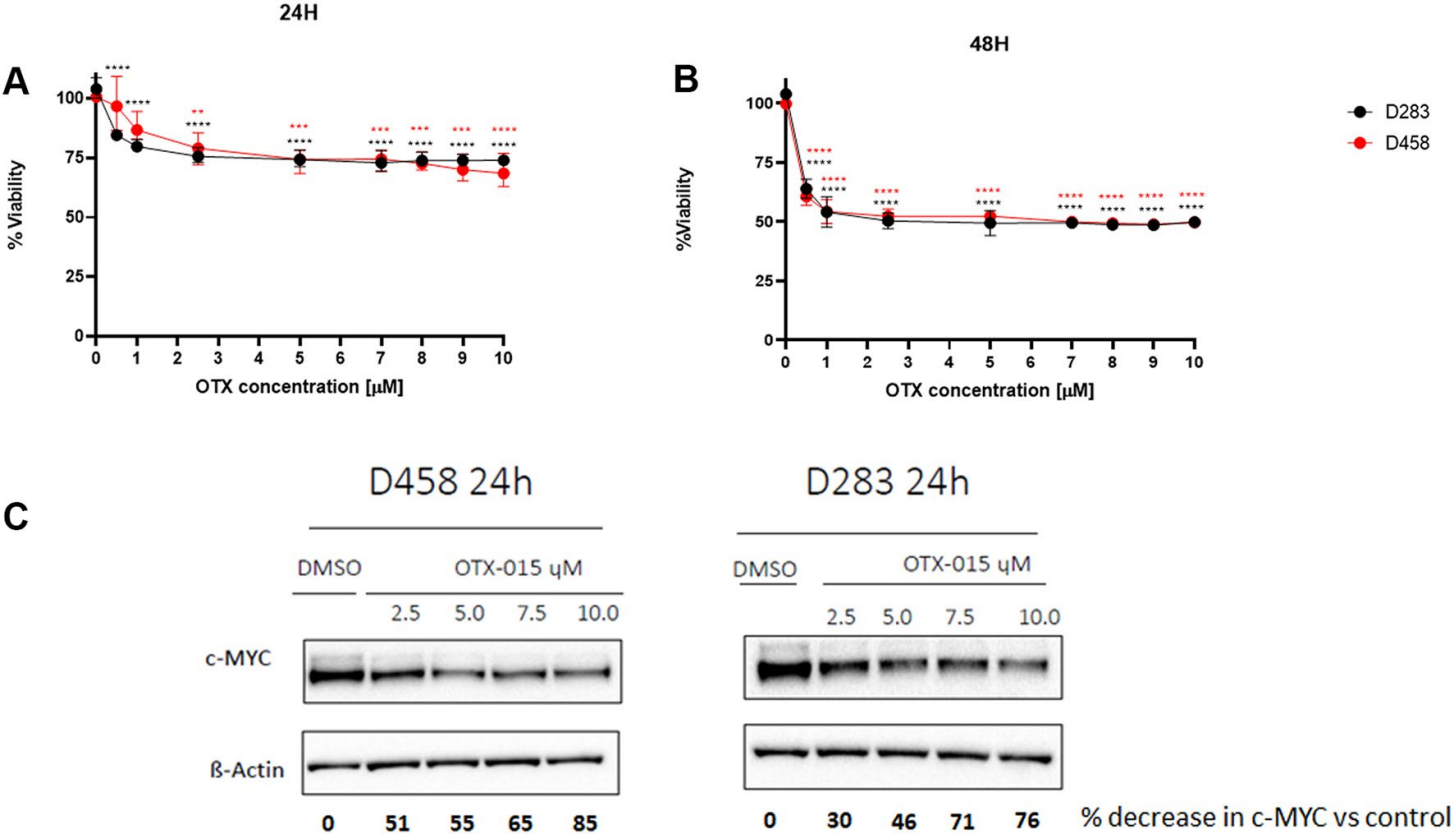
OTX-015 inhibits the viability of D458 and D283 MB cell lines. D458 (red) and D283 (black) were treated with different concentrations of OTX-015 spanning from 0 to 10 µM as indicated for (**A**) 24 and (**B**) 48 h. Statistical significance of cell viability inhibition was performed using one-way ANOVA with **; *** and **** indicating *p* < 0.05, < 0.005 and < 0.0001, respectively. (**C**) Representative Western blot showing MYC downregulation upon treatment with OTX-015 (2.5, 5.0, 7.5, and 10.0 µM) for 24 h. Bold numbers indicate percentage reduction of normalised c-MYC (c-MYC/β-Actin) versus control (DMSO). Original Western blots are presented in Supplementary Fig. 1.

The D283 and D458 cells were treated with a range of OTX-015 doses spanning from 0 to 10 µM for 24 and 48 h, to evaluate the optimal time point to study their metabolism. Western Blot analysis was performed to evaluate MYC protein expression (Fig. 1A–C and Supplementary Fig. S1). Using the MTT assay we demonstrated that OTX-015 decreases cell viability by 50% after 48 h, while Western Blot showed that c-MYC protein levels were already reduced after 24 h of incubation with OTX-015. Specifically, relative protein quantification showed that 7.5 µM of OTX-015 caused a 70% decrease in c-MYC protein levels *versus* untreated cells. We thus choose to investigate the early effects of BET inhibition and MYC downregulation on cancer metabolism after 24 h of incubation with 7.5 µM of OTX-015.

### OTX-015 treatment causes a statistically significant metabolic shift in MYC-driven MB cell lines

**Figure 2:**
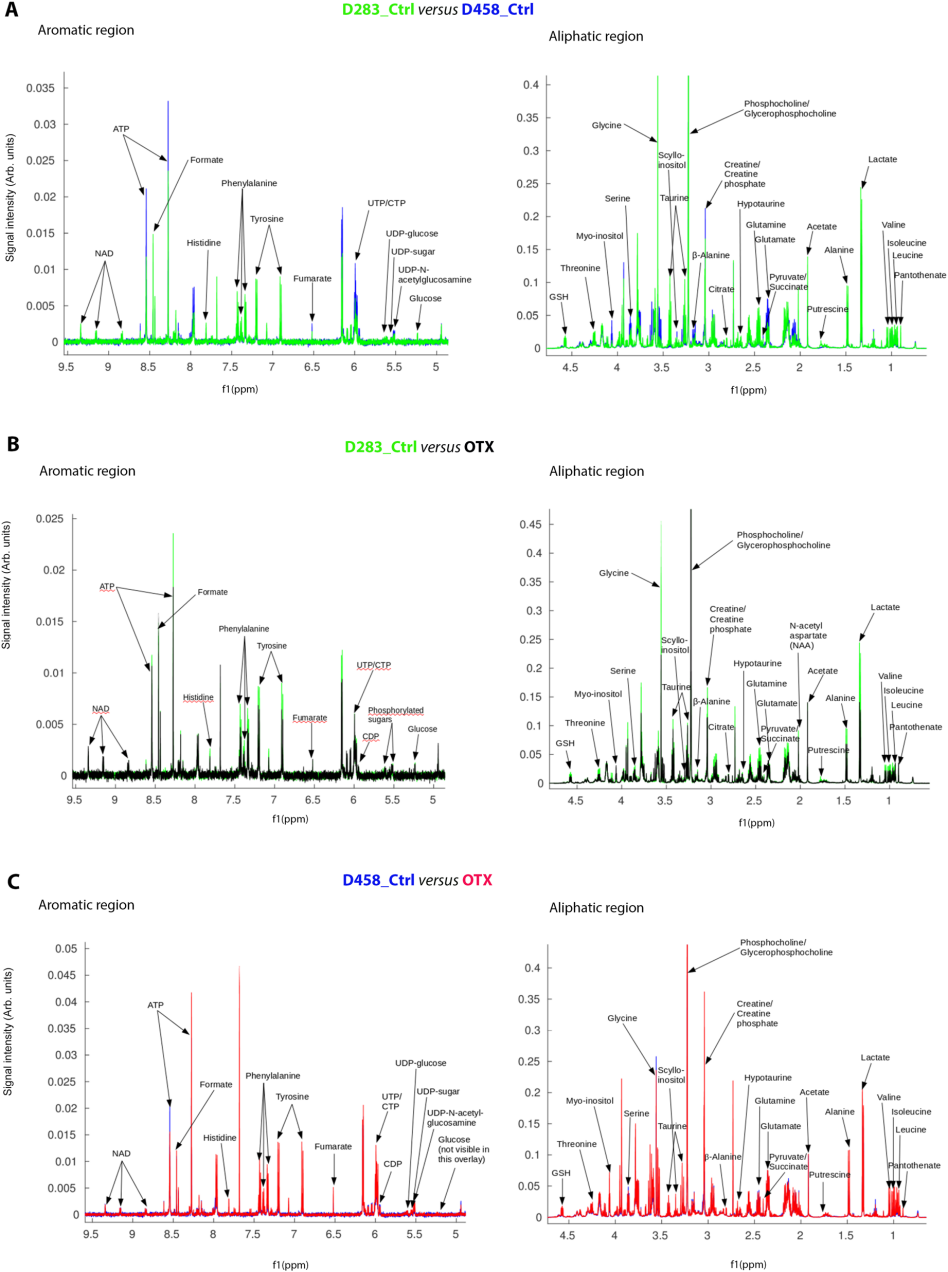
Aligned and normalised overlays of representative one-dimensional 600 MHz ^1^H NMR spectra of cellular extracts from control and OTX-015 treated cells, focussing on aliphatic and aromatic regions. (**A**) D283 (D283_Ctrl, green) versus D458 (D458_Ctrl, blue); (**B**) D283 control (D283_Ctrl, green) versus OTX-015 treated (D283_OTX, black) and (**C**) D458 control (D458_Ctrl, blue) versus OTX-015 treated (D458_OTX, red) cell lines. Selected peaks of identified metabolites are labelled.

Next, we analysed the metabolism of D283 and D458 cells upon OTX-015 treatment by Nuclear Magnetic Resonance (NMR) spectroscopy. ^1^H NMR profiling of the aqueous cellular extracts allowed the identification of 41 intracellular polar metabolites (Fig. 2, Supplementary Figs. S2–S7, and Supplementary Table S1), assigned by comparing peak chemical shifts, J-couplings and multiplet intensity ratios to NMR spectral resonance patterns available in the Human Metabolome Database (HMDB)^32^ and the biological magnetic resonance data bank (BMRB)^33^. Metabolite assignment was confirmed by a 2D NMR approach, performing [^1^H–^1^H] TOCSY and [^1^H–^13^C] HSQC experiments (Supplementary Figs. S8–S10). Aligned and normalised 1D ^1^H-NMR spectra overlays of the aliphatic and aromatic region of D283 *versus* D458, OTX-015-treated D283 (D283_OTX) versus D283 control (D283_Ctrl) and OTX-015-treated D458 (D458_OTX) versus D458 control (D458_Ctrl) (Fig. 2, and Supplementary Figs. S2–S7) show intensity differences for many metabolite peaks, the chemical shift values of which are reported in Supplementary Table S1. Amongst these we detected: glycolysis and tricarboxylic acid (TCA) cycle derivatives including lactate, acetate, alanine, succinate, citric acid, pyruvate, glucose and fumarate; glutamine and glutamine derivatives comprising glutamate *N*-acetylaspartate and proline; other amino acids such as leucine, isoleucine, valine, phenylalanine, threonine, tyrosine, glycine, serine and histidine; amino acid derivatives including reduced glutathione GSH, creatine, taurine, hypotaurine and formate; phospholipid derivatives including phosphocholine and glycerophosphocholine; nucleotides as ATP, UDP, CTP and NAD; the carbohydrate myo-inositol and its stereoisomer scyllo-inositol; the polyamine putresceine and the vitamin B5 pantothenate. Furthermore, two phosphorylated sugars were observed to change upon OTX-015 treatment. One of these was identified based on the chemical shift of the anomeric proton as UDP-N-acetylglucosamine, whilst the other was dubbed as a generic ‘UDP-sugar’, as the unambiguous assignment of its structure was not possible from our NMR data alone.

**Figure 3:**
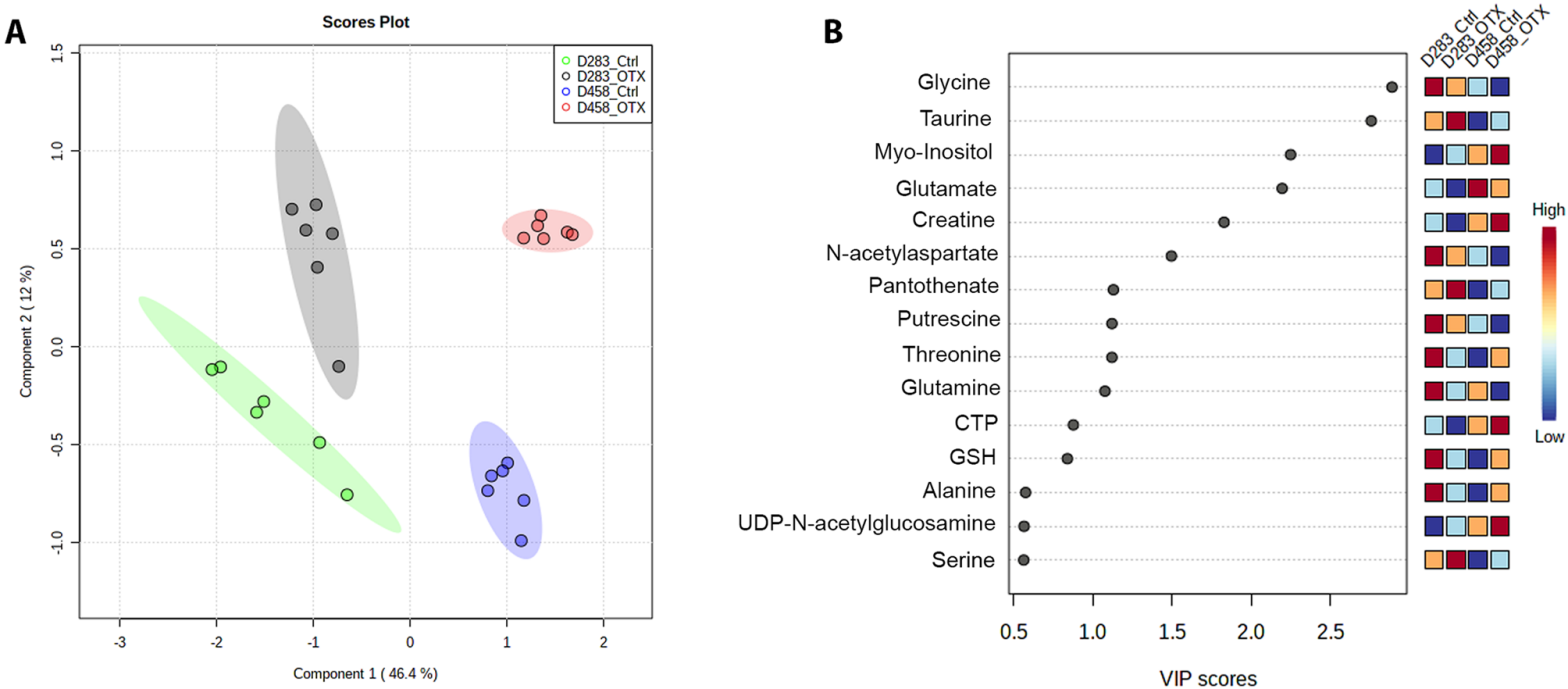
(**A**) Score scatter plot showing PLS-DA analysis of the full spectra of ^1^H NMR data, where peak intensities of metabolites represent the variables. The spatial distribution of the samples is based on the metabolite pattern. Six biological replicates were used for this analysis. (**B**) Variable importance of projection (VIP) score was plotted for the metabolites that significantly contributed (VIP ˃ 1) to separation by PLS-DA.

1D ^1^H NMR spectra were used to perform a Partial least squares discriminant analysis (PLS-DA), a supervised multivariate statistical analysis which is a powerful tool for dealing with large-scale metabolomics studies^34^. The PLS-DA score scatter plot shows that the metabolome of D283 and D458 cell lines is statistically different, with component 1 and component 2, with a variance of 46.4% and 12% respectively, responsible for group separation (Fig. 3A). Notably, a clear change of the metabolome is detectable in both cell lines upon the treatment with OTX-015, as D283_OTX and D458_OTX are shifted along component 2 compared to their respective controls (D283_Ctrl and D458_Ctrl). Next, to identify the metabolites that contribute to the observed PLS-DA separation, variable importance in projection (VIP) scores were determined for the NMR data set (Fig. 3B). VIP scores ˃1 suggest that dissimilar levels of glycine, taurine, myo-inositol, glutamate, creatine, and *N*-acetyl aspartate are strongly associated with the separation of D283 and D458 cells, whereas increased myo-inositol and taurine, and reduced glycine, glutamate and *N*-acetyl aspartate determine PLS-DA separation between treatment and controls.

### Key metabolites regulated by OTX-015 are shared in both MB cell lines

**Figure 4:**
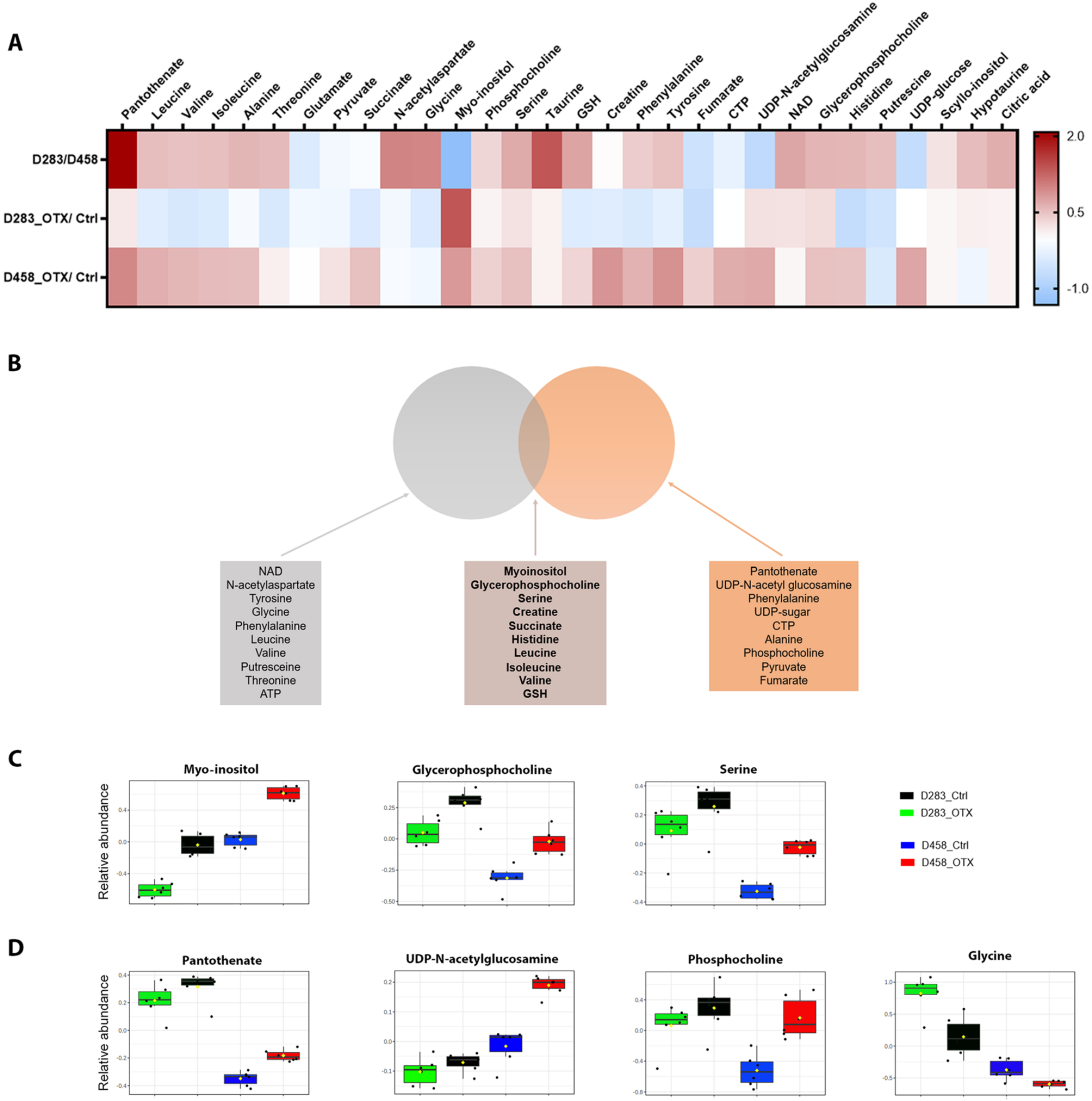
(**A**) Heatmap showing metabolite quantification (Log2 fold change values) in D283 versus D458 cell lines, D283_OTX versus D283_Ctrl and D458_OTX versus D458_Ctrl. Metabolites were quantified with MATLAB, according to intensity of metabolite signal in the ^1^H NMR spectrum and calculated using an internal standard (n = 6). (**B,C**) Boxes depicting metabolites commonly deregulated in D283_OTX versus D283_Ctrl and D458_OTX versus D458_Ctrl and statistically significant in both the comparisons (**B**) or at least in one of them (**C**). Statistical analysis has been performed using one way-ANOVA (see Supplementary Table 1).

A heat map representing a quantitative targeted analysis confirmed key metabolic differences between the groups identified by PLS-DA (Fig. 4A). One-way analysis of variance (ANOVA) for this metabolite quantification is presented in Table 1. Fisher's Least Significant Difference (LSD) indicates that D283 and D458 are characterised by different levels of amino acids and derivatives (*N*-acetylaspartate, taurine, hypotaurine, tyrosine, glycine, phenylalanine, serine, leucine, isoleucine, valine, alanine, GSH, glutamate, and threonine); carbohydrates (myo-inositol, scyllo-inositol, UDP-*N*-acetylglucosamine, and UDP-sugar); phospholipid derivatives (phosphocholine and glycerophosphocholine); nucleotides (NAD and CTP); the polyamine putresceine, and the vitamin B5 pantothenate.

**Table 1:** Features identified by one-way ANOVA and post hoc analysis using Metaboanalyst 5.0.

Metabolite	f.value	p.value	−log10(p)	FDR	Fishers LSD
Myo-inositol	142.29	1.17E−13	12.932	4.79E–12	D283_OTX–D283_Ctrl; D458_Ctrl–D283_Ctrl; D458_OTX–D283_Ctrl; D458_OTX–D283_OTX; D458_OTX–D458_Ctrl
NAD	89.231	9.44E−12	11.025	1.94E–10	D283_OTX–D283_Ctrl; D283_Ctrl–D458_Ctrl; D283_Ctrl–D458_OTX; D283_OTX–D458_Ctrl; D283_OTX–D458_OTX
Pantothenate	80.609	2.41E−11	10.617	3.22E–10	D283_Ctrl–D458_Ctrl; D283_Ctrl–D458_OTX; D283_OTX–D458_Ctrl; D283_OTX–D458_OTX; D458_OTX–D458_Ctrl
*N*-acetylaspartate	78.341	3.14E−11	10.503	3.22E–10	D283_Ctrl–D283_OTX; D283_Ctrl–D458_Ctrl; D283_Ctrl–D458_OTX; D283_OTX–D458_Ctrl; D283_OTX–D458_OTX
Taurine	68.362	1.09E−10	9.9632	8.92E–10	D283_Ctrl–D458_Ctrl; D283_Ctrl–D458_OTX; D283_OTX–D458_Ctrl; D283_OTX–D458_OTX
Creatine	59.695	3.69E−10	9.4327	2.52E–09	D283_Ctrl–D283_OTX; D458_OTX–D283_Ctrl; D458_Ctrl–D283_OTX; D458_OTX–D283_OTX; D458_OTX–D458_Ctrl
Tyrosine	56.716	5.83E−10	9.2343	3.26E–09	D283_Ctrl–D283_OTX; D283_Ctrl–D458_Ctrl; D458_OTX–D283_Ctrl; D283_OTX–D458_Ctrl; D458_OTX–D283_OTX; D458_OTX–D458_Ctrl
Hypotaurine	56.159	6.37E−10	9.1961	3.26E–09	D283_Ctrl–D458_Ctrl; D283_Ctrl–D458_OTX; D283_OTX–D458_Ctrl; D283_OTX–D458_OTX
UDP-*N*-acetylglucosamine	53.675	9.51E−10	9.0218	4.33E–09	D458_Ctrl–D283_Ctrl; D458_OTX–D283_Ctrl; D458_Ctrl–D283_OTX; D458_OTX–D283_OTX; D458_OTX–D458_Ctrl
Glycine	47.974	2.55E−09	8.5934	1.05E–08	D283_Ctrl–D283_OTX; D283_Ctrl–D458_Ctrl; D283_Ctrl–D458_OTX; D283_OTX–D458_Ctrl; D283_OTX–D458_OTX
Citric acid	45.963	3.70E−09	8.4316	1.38E–08	D283_Ctrl–D458_Ctrl; D283_Ctrl–D458_OTX; D283_OTX–D458_Ctrl; D283_OTX–D458_OTX
Succinate	42.472	7.32E−09	8.1356	2.50E–08	D283_Ctrl–D283_OTX; D458_OTX–D283_Ctrl; D458_Ctrl–D283_OTX; D458_OTX–D283_OTX; D458_OTX–D458_Ctrl
Glycerophosphocholine	34.849	3.90E−08	7.4091	1.23E–07	D283_OTX–D283_Ctrl; D283_Ctrl–D458_Ctrl; D283_OTX–D458_Ctrl; D283_OTX–D458_OTX; D458_OTX–D458_Ctrl
Phenylalanine	32.012	7.86E−08	7.1045	2.30E–07	D283_Ctrl–D283_OTX; D283_Ctrl–D458_Ctrl; D458_OTX–D283_Ctrl; D458_OTX–D283_OTX; D458_OTX–D458_Ctrl
UDP-sugar	29.259	1.63E−07	6.7869	4.46E–07	D458_Ctrl–D283_Ctrl; D458_OTX–D283_Ctrl; D458_Ctrl–D283_OTX; D458_OTX–D283_OTX; D458_OTX–D458_Ctrl
Histidine	25.497	4.88E−07	6.3117	1.25E–06	D283_Ctrl–D283_OTX; D283_Ctrl–D458_Ctrl; D458_OTX–D283_OTX; D458_OTX–D458_Ctrl
Serine	25.026	5.64E−07	6.2484	1.36E–06	D283_OTX–D283_Ctrl; D283_Ctrl–D458_Ctrl; D283_OTX–D458_Ctrl; D283_OTX–D458_OTX; D458_OTX–D458_Ctrl
Leucine	20.657	2.44E−06	5.6126	5.56E–06	D283_Ctrl–D283_OTX; D283_Ctrl–D458_Ctrl; D458_OTX–D283_OTX; D458_OTX–D458_Ctrl
CTP	19.627	3.56E−06	5.4482	7.69E–06	D458_Ctrl–D283_Ctrl; D458_OTX–D283_Ctrl; D458_Ctrl–D283_OTX; D458_OTX–D283_OTX; D458_OTX–D458_Ctrl
GSH	17.53	8.07E−06	5.093	1.66E–05	D283_Ctrl–D283_OTX; D283_Ctrl–D458_Ctrl; D283_Ctrl–D458_OTX; D283_OTX–D458_Ctrl; D458_OTX–D458_Ctrl
Isoleucine	16.708	1.13E−05	4.9454	2.12E–05	D283_Ctrl–D283_OTX; D283_Ctrl–D458_Ctrl; D458_OTX–D283_OTX; D458_OTX–D458_Ctrl
Valine	16.702	1.14E−05	4.9443	2.12E–05	D283_Ctrl–D283_OTX; D283_Ctrl–D458_Ctrl; D458_OTX–D283_OTX; D458_OTX–D458_Ctrl
Alanine	16.271	1.37E−05	4.8648	2.43E–05	D283_Ctrl–D458_Ctrl; D283_OTX–D458_Ctrl; D458_OTX–D458_Ctrl
Putrescine	12.322	8.68E−05	4.0613	0.00014834	D283_Ctrl–D283_OTX; D283_Ctrl–D458_Ctrl; D283_Ctrl–D458_OTX
Glutamate	11.426	0.00013918	3.8564	0.00022825	D458_Ctrl–D283_Ctrl; D458_OTX–D283_Ctrl; D458_Ctrl–D283_OTX; D458_OTX–D283_OTX
Phosphocholine	10.27	0.00026472	3.5772	0.00041744	D283_Ctrl–D458_Ctrl; D283_OTX–D458_Ctrl; D458_OTX–D458_Ctrl
Threonine	9.7105	0.00036679	3.4356	0.00055698	D283_Ctrl–D283_OTX; D283_Ctrl–D458_Ctrl; D283_Ctrl–D458_OTX
Pyruvate	9.4295	0.00043384	3.3627	0.00063527	D458_OTX–D283_Ctrl; D458_Ctrl–D283_OTX; D458_OTX–D283_OTX; D458_OTX–D458_Ctrl
Scyllo-inositol	9.1696	0.000508	3.2941	0.00071821	D283_Ctrl–D458_Ctrl; D283_Ctrl–D458_OTX; D283_OTX–D458_Ctrl; D283_OTX–D458_OTX
Fumarate	5.5076	0.0063516	2.1971	0.0086805	D458_OTX–D283_Ctrl; D458_OTX–D283_OTX; D458_OTX–D458_Ctrl
ATP	4.3404	0.016453	1.7837	0.021761	D283_Ctrl–D283_OTX; D458_Ctrl–D283_OTX; D458_OTX–D283_OTX

Fisher's least significant difference (LSD) shows significant pairwise comparisons of the conditions under study.

Upon treatment with OTX-015, a metabolic response was detected in both D283 and D458 cell lines and interestingly a few metabolites appear to be commonly regulated. These include myo-inositol, glycerophosphocholine, serine, pantothenate, UDP-*N*-acetylglucosamine, phosphocholine and glycine, all of which increased in both D283 and D458 after treatment (Fig. 4B–D). Notably, OTX-015 treatment induced additional changes in the endogenous metabolome of D283 and D458, albeit the specific alterations differ between the two cell lines (Fig. 4A and Supplementary Fig. S11).

### Metabolite set enrichment analysis indicates significantly enriched metabolic pathways in MYC-driven MB cells

**Figure 5:**
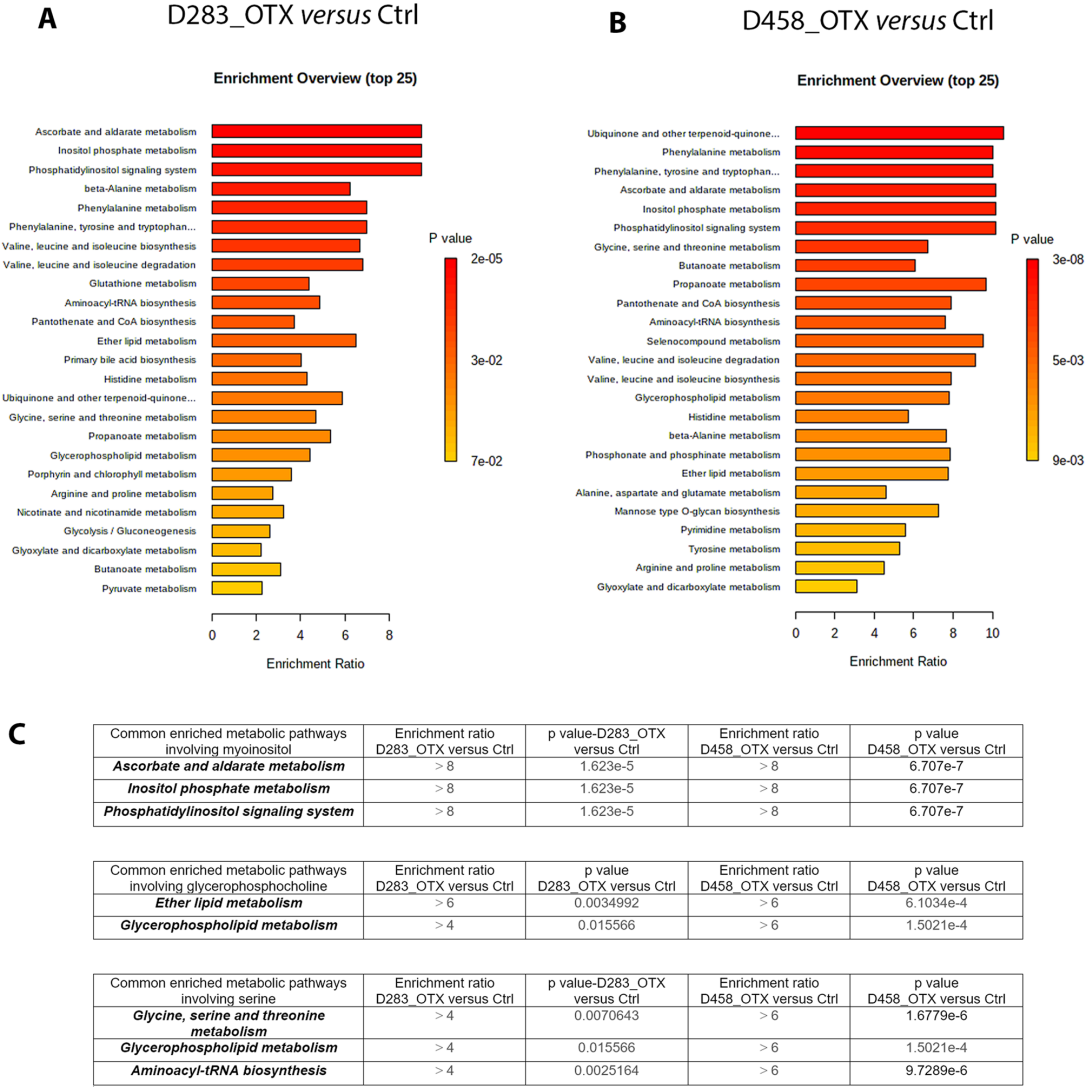
Summary plot of the metabolite set enrichment analysis (MSEA) of the identified metabolites in (**A**) D283_OTX *versus* D283_Ctrl and (**B**) D458_OTX *versus* D458_Ctrl. Horizontal graph bars indicate the pathways that are enriched in these groups. The colour code corresponds to the calculated *p*-value. Vertical red lines indicate pathways with a *p*-value < 0.05. (**C**) Summary table of the pathways commonly enriched in both the cell lines upon OTX-015 treatment and with a *p*-value < 0.05 are shown.

To understand how metabolic rewiring caused by the OTX-015 treatment impact on functional signalling pathways, we performed a metabolite set enrichment analysis (MSEA) on quantified metabolites (described in Table 1), along with their relative concentrations, using the web-based platform MetaboAnalyst^35^ (Fig. 5). The top 25 pathways significantly enriched in D283_OTX *versus* untreated control and D458_OTX *versus* untreated control cells are shown in Fig. 5A,B. We focused on the common enriched pathways in which the metabolites myo-inositol, glycerophosphocholine and serine increased significantly upon OTX-015 treatment in both cell lines (Fig. 5).

These data confirm that OTX-015 triggers a different metabolic reprogramming in D283 and D458 MB cell lines, reflecting their diverse basal metabolism. Nonetheless, in both cell lines OTX-015 affects ascorbate and aldarate metabolism, inositol phosphate metabolism, phosphatidylinositol signalling system, ether lipid metabolism, glycerophospholipid metabolism, glycine serine and threonine metabolism and aminoacyl tRNA biosynthesis (Fig. 5C).

## Discussion

MYC-driven MB is an aggressive prevalent paediatric tumour, which is refractory to intensive multimodal therapy^4,36^. In this scenario, BETis are emerging as promising agents in MYC-dependent MB as well as in other tumours, because of their ability to impair MYC expression and to modulate the epigenetic machinery of cancer cells^4,20^. In this work, we studied the effect of the BETi OTX-015 on the metabolism of the D283 and D458 MYC-driven childhood MB cell lines. OTX-015 has been developed from the conventional BETi JQ1 to improve its pharmacokinetic profile and thus its potential in clinical use^16,17,22^.

It is already known that MYC regulates metabolism which in turn controls MYC levels^27^. MYC promotes cell proliferation and induces metabolic alterations because of increased anabolic demand. On the other hand, MYC is also downstream of multiple control mechanisms regulated by nutrient levels and metabolic stress^27^. Despite the well-recognised crosstalk between MYC and metabolism in cancer, how metabolism is deregulated in MYC-driven MB is still unknown. Furthermore, although inhibition of BRD proteins is known to cause metabolic reprogramming^37,38^, whether BETi-dependent metabolic changes can be used to monitor drug response towards BETis is not understood. Here, we investigated the metabolome of MYC-driven MB cell models and interrogated how OTX-015 would modulate their metabolic phenotype using an NMR-based approach. NMR spectroscopy has proven to be of high value as an accurate and quantitative method to investigate metabolic alterations related to disease, through detecting metabolic signatures for early diagnosis and prognosis evaluation, unveiling cellular mechanisms and discovering pharmacological actions and potential therapeutic significance of drug compounds^39–41^.

In this work, we discovered that D283 exhibits diverse metabolic characteristics compared to D458 cells, consistent with their different origin and genetic background^28^. The most significantly different metabolites between the two cell lines were: amino acids and their derivatives such as hypotaurine and GSH; the TCA cycle metabolite citric acid; the phospholipid derivatives phosphocholine and glycerol-phosphocholine; the vitamin B5 pantothenate; NAD and scylloinositol. Upon treatment of D283 and D458 with OTX-015, common features were revealed in addition to cell-specific metabolic changes. In both cell lines, quantitative NMR data showed an increase in myo-inositol, a metabolite well known to control cell osmoregulation, whose role in pathophysiological contexts is tissue-and tumour-dependent^42^. Notably, myo-inositol targets critical cancer liabilities^43–45^ by: (i) inhibiting pRB phosphorylation and subsequently blocking the cell cycle; (ii) reducing PI3K, Akt, ERK and NF-kB and (iii) impairing invasiveness^43–45^. The observed increase in myo-inositol levels therefore may contribute to the anti-proliferative effect observed in D283 and D458 cell lines upon treatment with OTX-015.

Metabolite set enrichment analysis (MSEA) revealed that OTX-015 treatment causes a marked common enrichment in metabolic pathways involving myo-inositol (enrichment ratio ˃8). These include ascorbate and aldarate metabolism, inositol phosphate metabolism and phosphatidylinositol signalling system pathways in both cell lines. To our knowledge, ascorbate and aldarate metabolism has never previously been linked to MB, albeit it has been described as markedly altered in other cancers^46^. Notably, both ascorbate (AA) and its oxidised form dehydroascorbate (DHA), which together represent the two existing redox states of Vitamin C, are important for neural function and antioxidant/oxidant balance^47^. Indeed, aberrant doses of DHA induce oxidative effects selectively killing cancer cells^48^. In agreement with previous work^49^, our results suggest that, beyond MYC downregulation, OTX-015, and perhaps other BETis, could elicit their antitumour capabilities also by disrupting the metabolic equilibrium between antioxidants and ROS, thereby eventually leading to cancer cell death.

Our data also show that inositol phosphate metabolism and phosphatidylinositol signalling system pathways are associated with OTX-015 treatment. These two pathways are well known to be initiated upon stimulation of phospholipase C, which metabolises phosphatidylinositol 4,5-bisphosphate into the intracellular second messengers 1,2-diacylglycerol (DAG) and inositol 1,4,5-trisphosphate (IP3)^50^. DAG activates protein kinase C, whilst IP3 regulates cellular calcium, and both can be converted into more polar inositol phosphates (IPs) and diphosphoryl inositol phosphates (PP-IPs). Inositols, of which the most abundant stereoisomer is myo-inositol^51^, play a crucial role in several biological functions and deregulation of their synthesis has been linked to disease including cancer^43,44^. IPs and PP-IPs are key in transcription control, mRNA export and DNA repair, and are also important for glucose sensing in β-pancreatic cells^52^. As mentioned above, inositols can impair oncogenic signalling using several mechanisms including inhibition of the PI3K/Akt pathway^43^. Notably, this has already been recognised in medulloblastoma as one of the relevant oncogenic pathways contributing to the disease^53^ and, accordingly, a selective pan-class I PI3K inhibitor namely BKM120 has been revealed as a potential novel therapy for MB^54^. Thus, in our MYC-driven cell models OTX-015 treatment may upregulate myo-inositol production, counteracting PI3K/Akt/mTOR signalling and eventually triggering tumour cell death.

Our NMR analysis recorded higher concentrations of glycerophosphocholine and phosphocholine upon OTX-015 treatment. Increase in these metabolites has been strongly associated with the well-known accumulation of lipid droplets^55,56^. Previous work demonstrated that MYC inhibition, achieved either with the c-MYC/Max inhibitor 10058-F4 or by MYC depletion, triggers mitochondrial dysfunction and β-fatty acid oxidation impairment, eventually leading to accumulation of intracellular lipid droplets^57,58^. Notably, BRD4 inhibition has also been shown to regulate OXPHOS capacity and to rewire metabolism in complex I (CI) mutant cybrid cells^37^. Consistently with this, our data appear to suggest that one of the putative anticancer mechanisms of OTX-015 may be hindering mitochondrial function, which would lead to inefficient lipid catabolism and increased levels of glycerophosphocholine and phosphocholine. Further experiments are however needed to investigate the intriguing possibility of a crosstalk between BETis, the mitochondrial machinery and lipid metabolism in MYC-driven MB.

In agreement with the increased levels of glycerophosphocholine, our MSEA analysis revealed that OTX-015 also causes a common enrichment of two intertwined metabolic pathways involving this metabolite, specifically ether lipid metabolism and glycerophospholipid metabolism. Although diverse roles of these pathways in cancer have been reported^59–62^, certain types of ether lipids function as endogenous antioxidants and are involved in cell differentiation^63^, whilst glycerophospholipids have been found decreased in glioma stem-like cells^64^. Changes in these two pathways may therefore have an impact on the grade of cell differentiation of MYC-driven MB.

Upon treatment with OTX-015, we also detected a significant concentration rise of serine in both cell lines. This amino acid is synthesised from the glycolytic intermediate 3-phosphoglycerate and can be transformed into glycine, which in turn is used in one carbon metabolism processes to sustain the synthesis of macromolecule building blocks such as purines, and/or for GSH synthesis to counteract ROS effects^65^. Importantly, serine-glycine conversion is catalysed by the serine hydroxymethyltransferase (SHMT), of which both the cytoplasmic (SHMT1) and mitochondrial (SHMT2) isoforms are known to be upregulated by MYC^66^. Thus, we could speculate that downregulation of MYC caused by OTX-015 impairs SHMT1/2 activity, resulting in serine accumulation. Consistent with this, we also detected a concomitant reduction in glycine in both cell lines and revealed that a commonly affected pathway following OTX-015 treatment is glycine, serine and threonine metabolism. Importantly, glycine is a precursor of purines and thus a key metabolite for rapidly proliferating cancer cells^67^. Accordingly, a recent study unveiled that glycine levels are increased in MYC-amplified MB with respect to the normal cerebellum^68^.

Another pathway that is affected in both cell lines in response to OTX-015 treatment is aminoacyltRNA biosynthesis. Notably, supporting our results, aminoacyltRNA biosynthesis has been reported as a pathway signature for group 3γ MB tumours, which is characterised by increased *MYC* copy number and negative prognosis^69^. AminoacyltRNA synthases are key enzymes in protein synthesis that during evolution acquired further domains, which provided these enzymes with additional roles beyond translation. Upregulation of seryl-tRNA synthetase induced cellular senescence and inhibited the growth of cervical tumour xenografts in mice by causing the senescence of tumour cells^70,71^. We found that MYC-driven MB cells treated with OTX-015 have significant higher levels of serine compared to untreated cells, and this is compatible with an upregulation of seryl-tRNA synthetase^70^. OTX-015 may therefore promote senescence by enhancing seryl-tRNA synthetase biosynthesis, although any link between OTX-015 and seryl-tRNA biosynthesis awaits to be proven.

In summary, in this work we describe the metabolic profile of MYC-driven MB cell models and reveal key metabolic changes triggered by OTX-015 treatment that could be either MYC-dependent or -independent. These novel insights contribute to the phenotypical characterisation of MYC-driven MB and could lay the foundation for uncovering new druggable signalling pathways beyond MYC-inhibition. Notably, our analysis revealed how OTX-015 was able to alter the metabolism of MYC-driven MB cell models. This provides key additional information on possible mechanisms of actions elicited by this compound at the cellular level to inhibit the growth of susceptible cancer cells, whilst also generating potentially useful clues for the use of BETis in the treatment of medulloblastoma. This first metabolic characterisation is likely to promote, guide and pave the ways to further analyses, which will likely reveal additional metabolic features and possible ramifications for the medulloblastoma treatment with OTX-015 or other BETis.

## Materials and methods

### Cell lines

The D283 and D458 cell lines were kindly provided by Associate Professor JI Johnsen (Karolinska Institutet, Stockholm, Sweden). Cells were maintained in DMEM supplemented with 10% heat-inactivated fetal bovine serum (FBS) (Life Technologies, Waltham, MA, USA), 10 mM Hepes, 2 mM L-glutamine, 100 IU/ml penicillin G, and 100 μg/ml streptomycin Sigma, St. Louis, MO, USA) at 37 °C in a humidified 5% CO2 atmosphere.

### Western blot

Whole-cell lysates were prepared using RIPA buffer (Sigma-Aldrich) supplemented with Halt Protease and Phosphatase Inhibitor Mixture (Thermo Scientific). Samples were resolved on SDS/PAGE and transferred to Polyvinylidene fluoride (PVDF) membranes (BioRad, 1704156). The c-Myc antibody (9E10) (NB600-302, Novus Biologicals Bio-Techne, United Kingdom 1:2000) was used to identify c-MYC, while anti-β-actin (Santa Cruz, SC47778 1:10,000) was used as loading control. HRP-conjugated secondary antibodies were from GE Healthcare. Bands were detected using SuperSignal West Dura chemiluminescent substrate (Thermo Fisher Scientific, 34075) and images were obtained in a ChemiDoc XRS + (BioRad). Image processing was performed with ImageLab (Life Science Research, BioRad). All Western blot data are the result of three independent biological replicates. The quantification was performed by normalising to β-actin and the percentage of decrease in c-MYC in treated *versus* control cells was calculated.

### Cell viability assay

Cell viability was evaluated by 3-(4,5-dimethylthiazol-2-yl)-2,5-diphenyltetrazolium bromide (MTT) reduction assay (Sigma-Aldrich). Cells were seeded in 96-well plates at a density of 135.000 cells/mL. Twenty-four hours after seeding, either drug (OTX-015, Sigma-Aldrich) or vehicle (DMSO, Sigma-Aldrich) was added to the media. After 48 h, 10 μL of MTT solution 5 mg/mL (Sigma-Aldrich) was added to each well. Two hours later, MTT was dissolved adding SDS 10% 0.01 M HCl and incubated overnight. The next day, absorbance at 570 nm was measured using a microplate reader (SpectraMax i3x Multi-Mode Microplate Reader, Molecular Devices). Experiments were repeated at least three times and viability was normalised to mean of untreated control, set as 100% viability.

### NMR experiment and analysis

#### Cell culture and extraction

Cells were seeded at a density of 5 × 10^6^ cells/mL in 150 mm dishes and incubated the next day with 7.5 µM OTX-015. After 24 h, cells were collected in 50 mL falcon tube, immerged in a dry ice/ethanol bath for 30 s and then centrifuged at + 4 °C at 200 g for 5 min. Media was discarded and the pellet suspend by adding 2.0 mL of -20 °C-cold MeOH. Afterwards, 2 mL of ice cold CH_3_Cl and MilliQ H_2_O were added and the solution was mixed by inversion and incubated on ice for 10 min to allow the formation of a stable bilayer. Subsequently, samples were centrifuged at + 4 °C at 300*g* for 45 min to separate the top aqueous layer from the bottom organic layer, containing metabolites soluble in the polar and apolar fraction, respectively. The polar fraction was dried in an Eppendorf concentrator (Concentrator plus, Eppendorf) and analysed by NMR.

#### NMR acquisition, processing and statistical analysis

Prior to NMR experiments, samples were resuspended in a 90/10 H_2_O/D_2_O buffer, with 100 mM Na_2_HPO_4_, 5 mM Trimethylsilylpropanoic acid (TSP) and 4 mM NaN_3_. NMR spectroscopy was performed on a Bruker Avance NEO 600 MHz NMR spectrometer equipped with a TCI CryoProbe Prodigy (Bruker). Spectra were acquired at 298 K and consisted for each sample of a 1D ^1^H PURGE spectrum^72^, which was then phase corrected, baseline corrected. The chemical shifts were referenced to the TSP peak at 0.0 ppm.

Spectra were then exported to MATLAB, using a toolbox written by A. Edison and co-workers^73^. Spectra were aligned by the Pearson PAFFT method (peak alignment by fast Fourier transform) and then normalised by probabilistic quotient normalisation (PQN).

Six biological replicates were used for this analysis.

#### Metabolite identification and quantification

2D spectra were used on one of the samples for identification purposes, consisting of a Total Correlation SpectroscopY ([^1^H–^1^H] TOCSY), using the Bruker pulse sequence “dipsi2gpphzs”, slightly modified to include presaturation, with eight scans, 256 t_1_ increments, a spectral width of 13.7 ppm in both dimensions and a relaxation delay of 2 s, along with a Heteronuclear Single-Quantum Correlation (([^1^H–^13^C] HSQC) spectrum, using the Bruker pulse sequence “hsqcetgpsisp”, with 16 scans, 256 t_1_ increments, a spectral width of 170 ppm in the ^13^C dimension and 12 ppm in the ^1^H dimension, and a relaxation time of 2 s. Metabolites were assigned using information acquired from the human metabolome database (HMDB)^32^ and the biological magnetic resonance data bank (BMRB)^33^. Relative quantification of metabolites was achieved by integration of peaks to calculate peak area, which is proportional to the number of nuclei that give rise to the signal. Mean ± SD values of peak areas were calculated for each metabolite.

#### Statistical analysis using MetaboAnalyst

After identification of the metabolites, a list of representative peaks was chosen for each of the identified metabolites (Supplementary Table 1). Their peak area for each spectrum was taken and stored in a table which, as a .csv file, was then loaded onto MetaboAnalyst^35^ for the statistical analysis. For multivariate analyses, spectra were scaled by the pareto method^74^. To determine whether spectra cluster into groups, PCA and PLS-DA with k-fold cross-validation were performed, along with a one-way ANOVA to see which metabolites were statistically different for specific group comparison.

#### Metabolic pathways enrichment analysis using MetaboAnalyst

The signal integrals corresponding to the identified metabolites were used to perform a Quantitative Enrichment Analysis employing MetaboAnalyst 5.0^35^ platform with the KEGG library, a database of relevant metabolic pathways found in humans^75–77^. Significant pathways were those with *p*-value < 0.05. The top 25 enriched pathways were shown.

## Supplementary Information

Supplementary Information.

## Data Availability

A large part of data generated or analysed during this study are included in this published article and its supplementary information files. Some of the datasets used and/or analysed during the current study have not been included and are available from the corresponding authors on reasonable request.
