# Supplementary material for: Metabolic rewiring in MYC-driven medulloblastoma by BET-bromodomain inhibition
Source: Sci Rep. 2023 Jan 23;13:1273. doi: 10.1038/s41598-023-27375-z (PMC9870962; doi:10.1038/s41598-023-27375-z)
Supplement: Supplementary file 1 — Supplementary Information. [file 41598_2023_27375_MOESM1_ESM.docx]

**Metabolic rewiring in MYC-driven medulloblastoma by BET-bromodomain inhibition**

Vittoria Graziani^1,4^, Aida Rodriguez Garcia^1^, Lourdes Sainero Alcolado^1^, Adrien Le Guennec^2^, Marie Arsenian Henriksson^1^* and Maria R Conte^3^*

*Co-corresponding authors

^1^Department of Microbiology and Tumor Biology, Biomedicum B7, Karolinska Institutet, SE-171 65 Stockholm, Sweden, ^2^Centre for Biomolecular Spectroscopy, King’s College London, Guy’s Campus, London SE1 1UL, United Kingdom, ^3^Randall Centre for Cell and Molecular Biophysics, King’s College London, Guy’s Campus, London SE1 1UL, United Kingdom

^4^Present address: Barts Cancer Institute, Queen Mary University of London, John Vane Science Building, Charterhouse Square, London EC1M 6BQ, UK

Correspondence to Maria R. Conte: [sasi.conte@kcl.ac.uk](mailto:sasi.conte@kcl.ac.uk) and Marie Arsenian Henriksson: marie.arsenian.henriksson@ki.se

Key words: medulloblastoma, OTX-015, metabolism, BET-bromodomain inhibitors

SUPPLEMENTARY MATERIAL


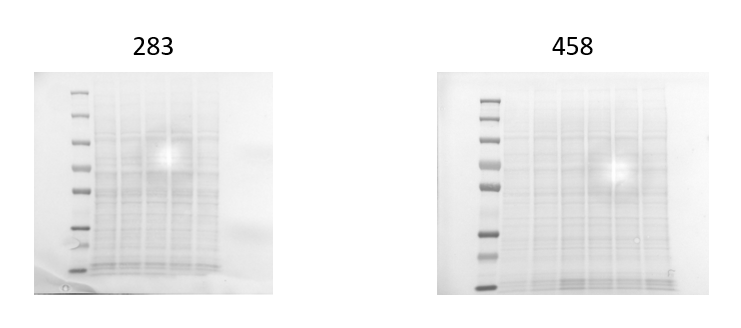


**A**


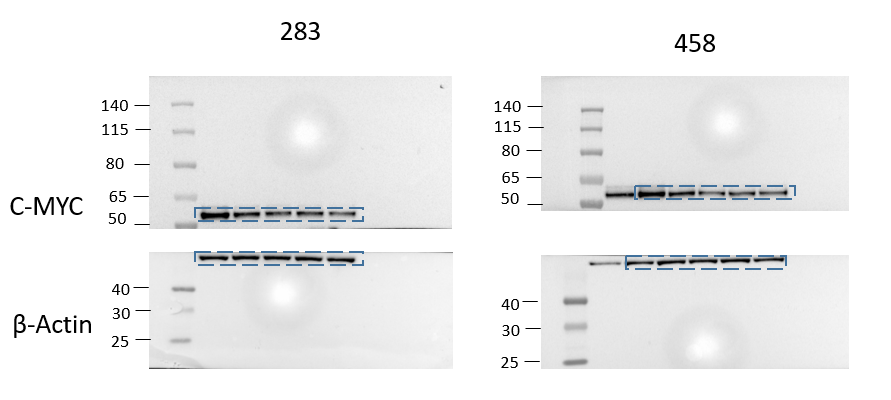


**B**

**C**


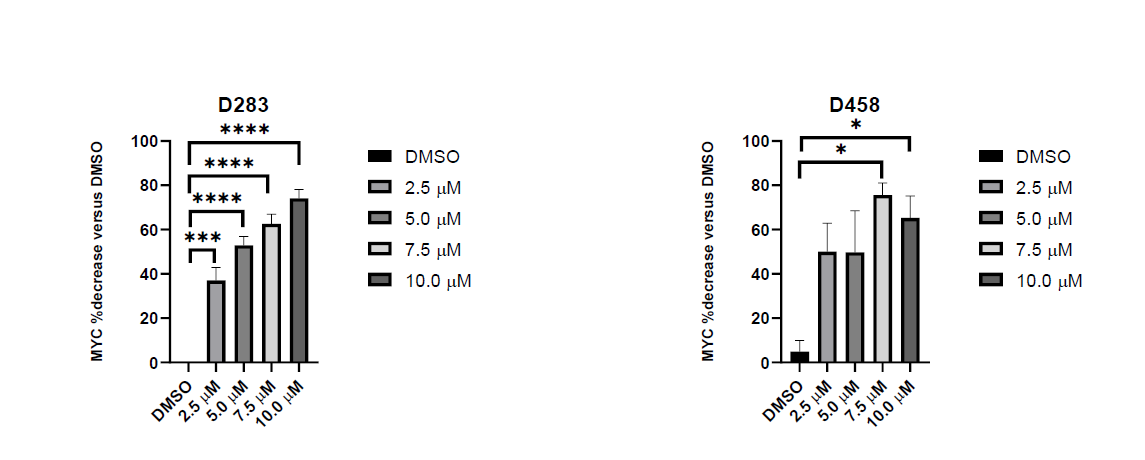


**Supplementary Figure S1.** (A) Original Western blots showing full length membranes of the trimmed panels of Figure 1, stained with Ponceau S. (B) Original blots upon hybridizations with anti C-MYC and anti-β-actin antibodies. The blots have been cut according to the molecular weight of the proteins of interest to allow multiple and simultaneously hybridizations, including with the anti-β-actin antibody that was used to normalise the signals. (C) Western blotting quantification from three independent biological replicates. Statistical analysis was performed using Ordinary one-way ANOVA and multiple comparisons, with **; *** and **** indicating p <0.05, <0.005 and <0.0001, respectively.


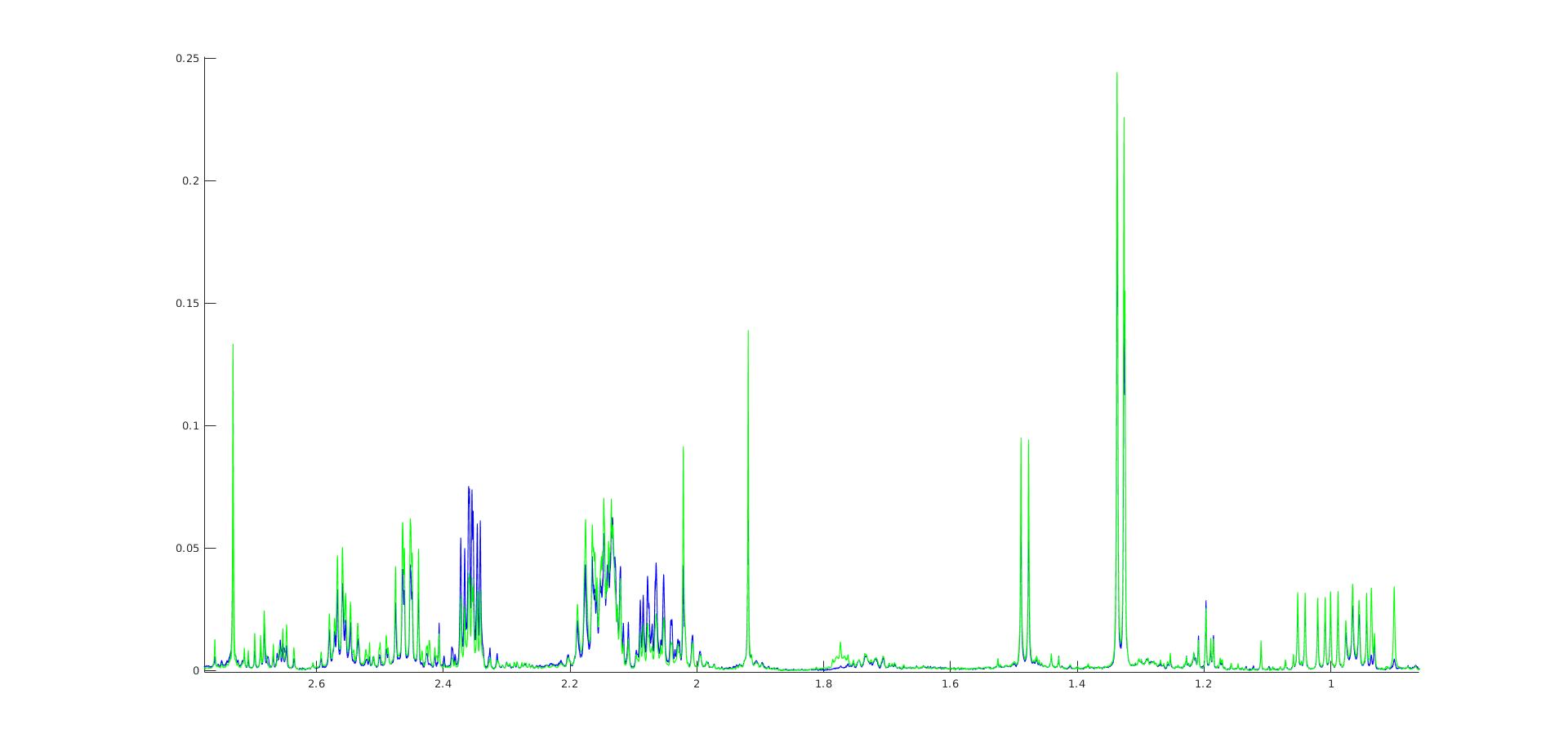


**A**


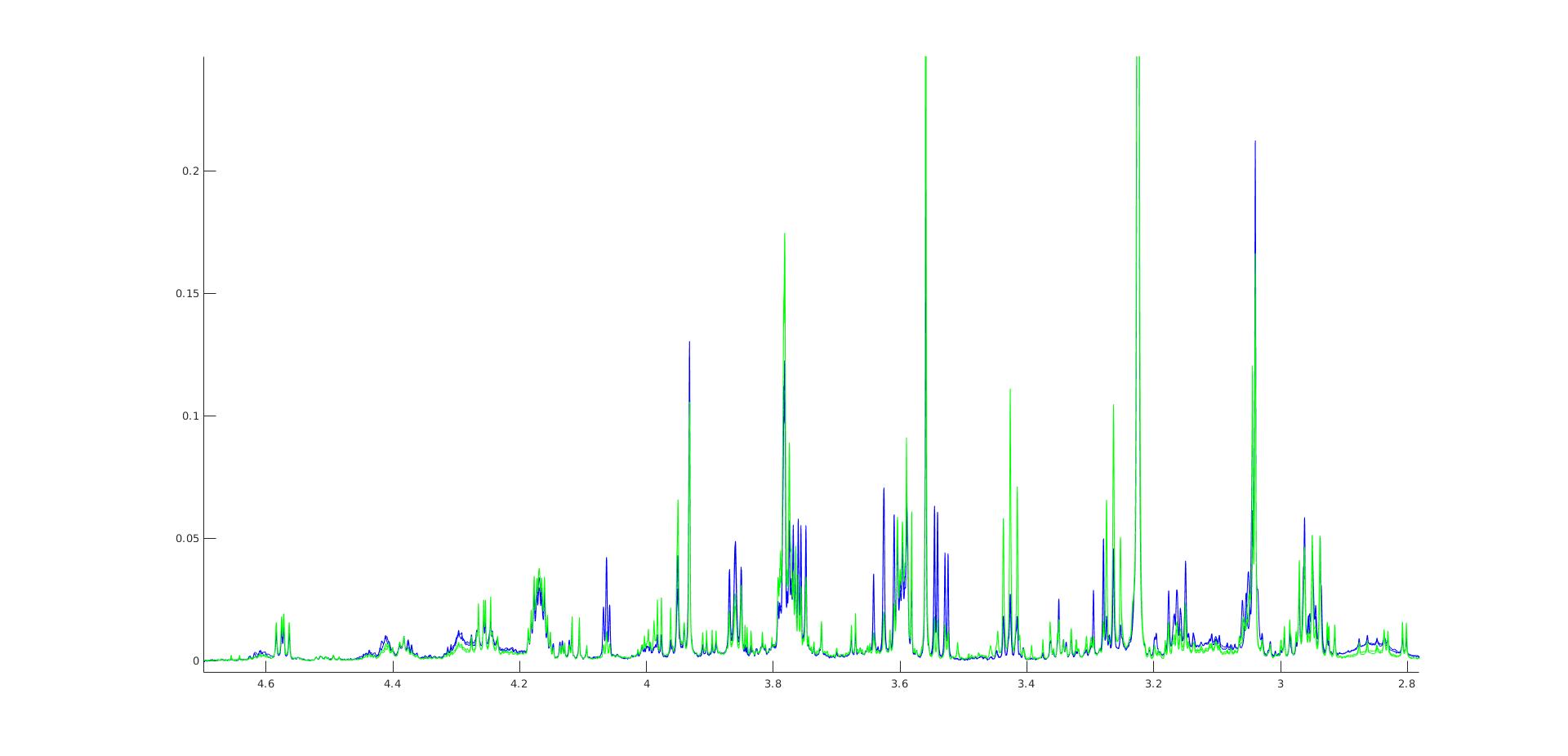


**B**

**Supplementary Figure S2.** Zoomed in part of the ^1^H NMR spectra representing D283_Ctrl versus D458_Ctrl overlay with D283_Ctrl in green and D458_Ctrl in blue. The panels A and B depict the following zoomed in regions: 1-3 ppm and 3-5 ppm, respectively.


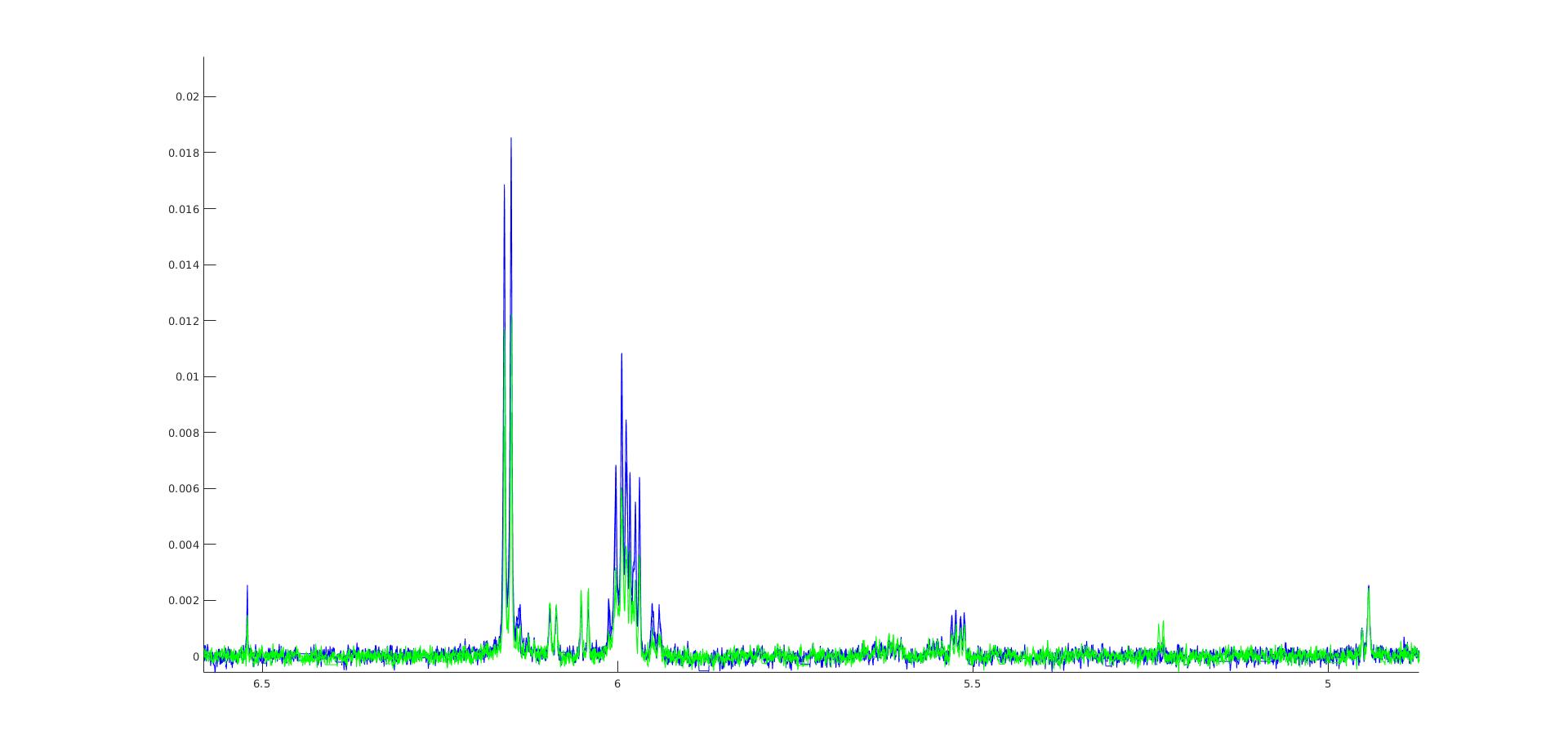


**A**


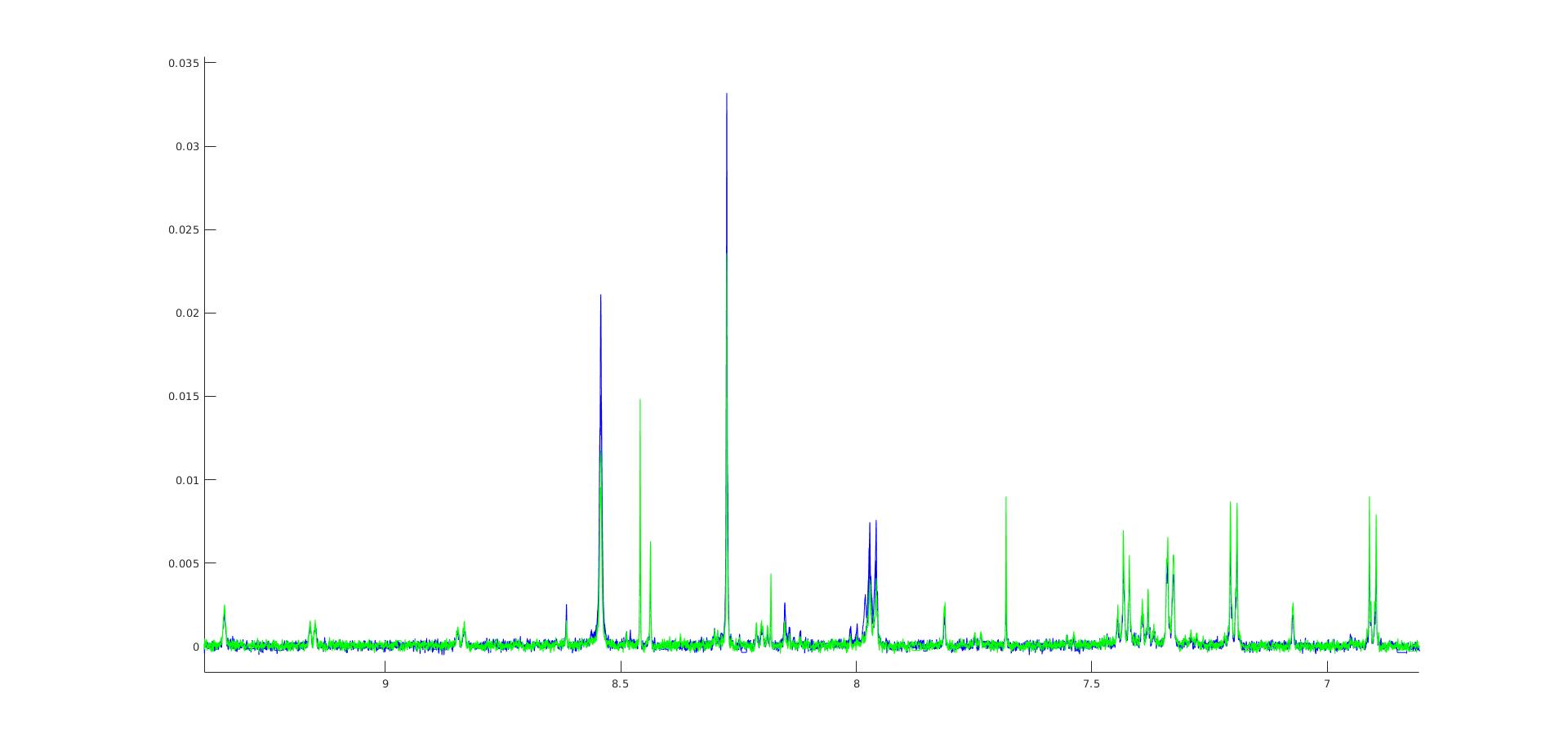


**B**

**Supplementary Figure S3.** Zoomed in part of the ^1^H NMR spectra representing D283_Ctrl versus D458_Ctrl overlay with D283_Ctrl in green and D458_Ctrl in blue. The panels A and B depict the following zoomed in regions: 5-7 ppm and 7-9 ppm, respectively.


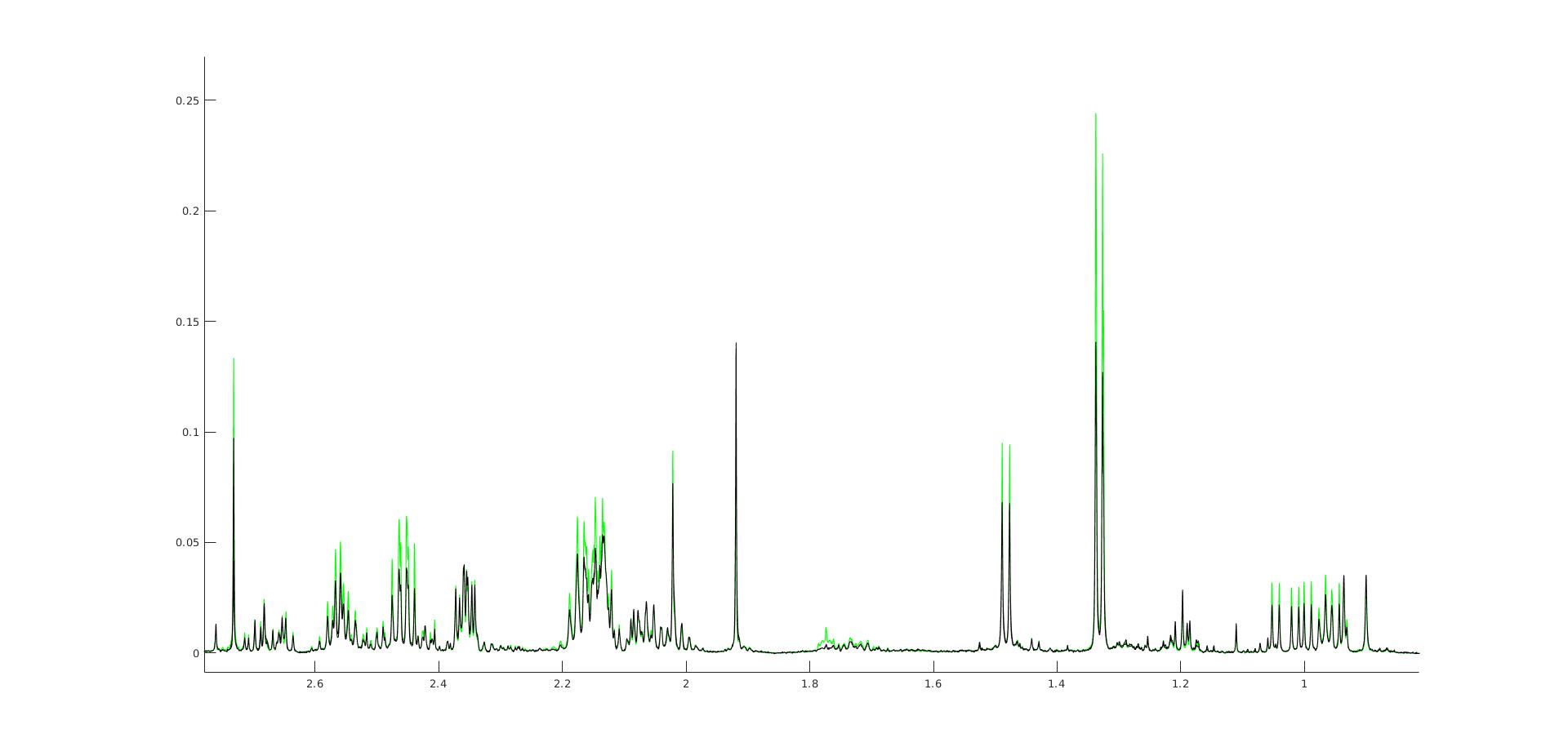


**A**


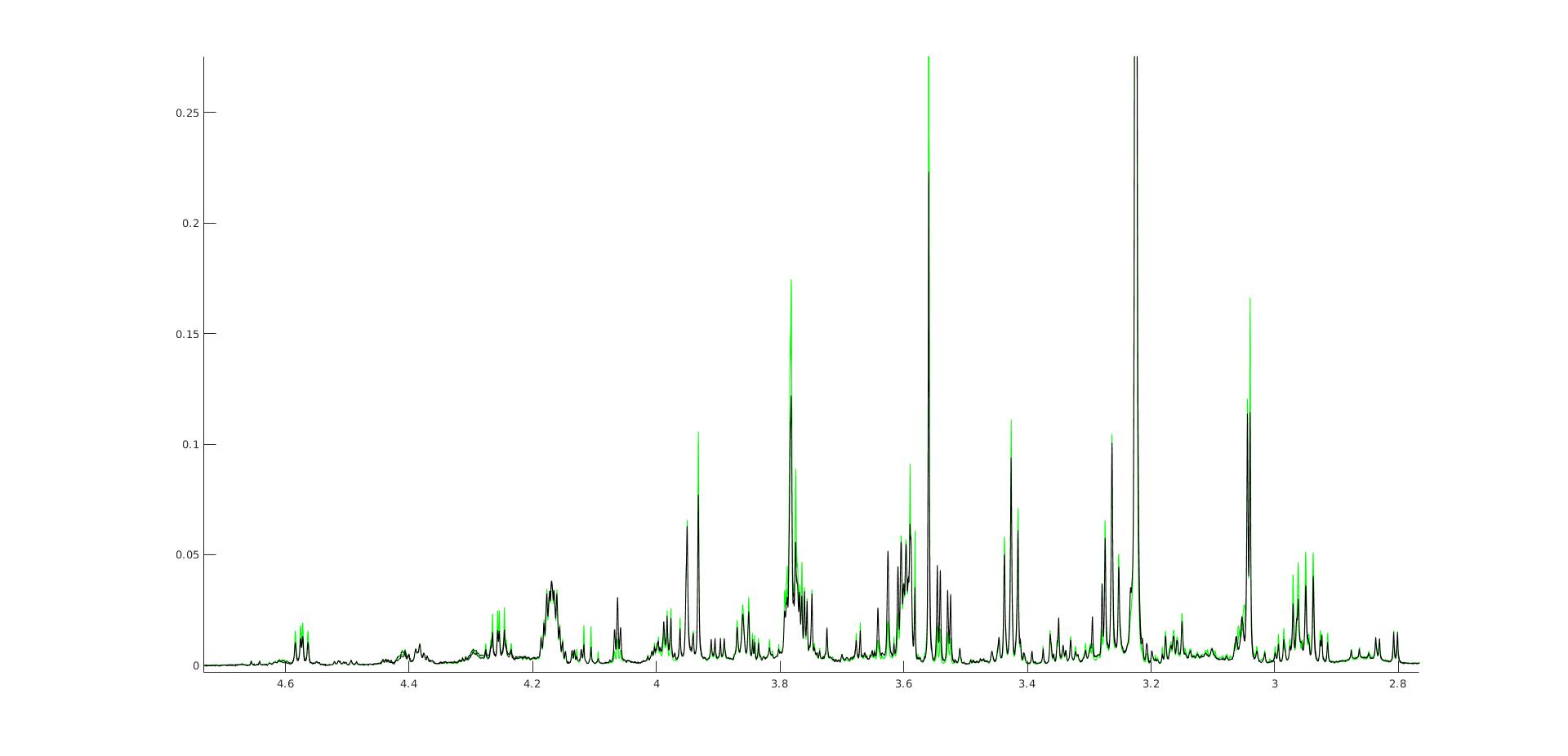


**B**

**Supplementary Figure S4.** Zoomed in part of the ^1^H NMR spectra representing D283_OTX versus Ctrl overlay with D283_OTX in black and D283_Ctrl in green. The panels A and B depict the following zoomed in regions: 1-3 ppm and 3-5 ppm, respectively.


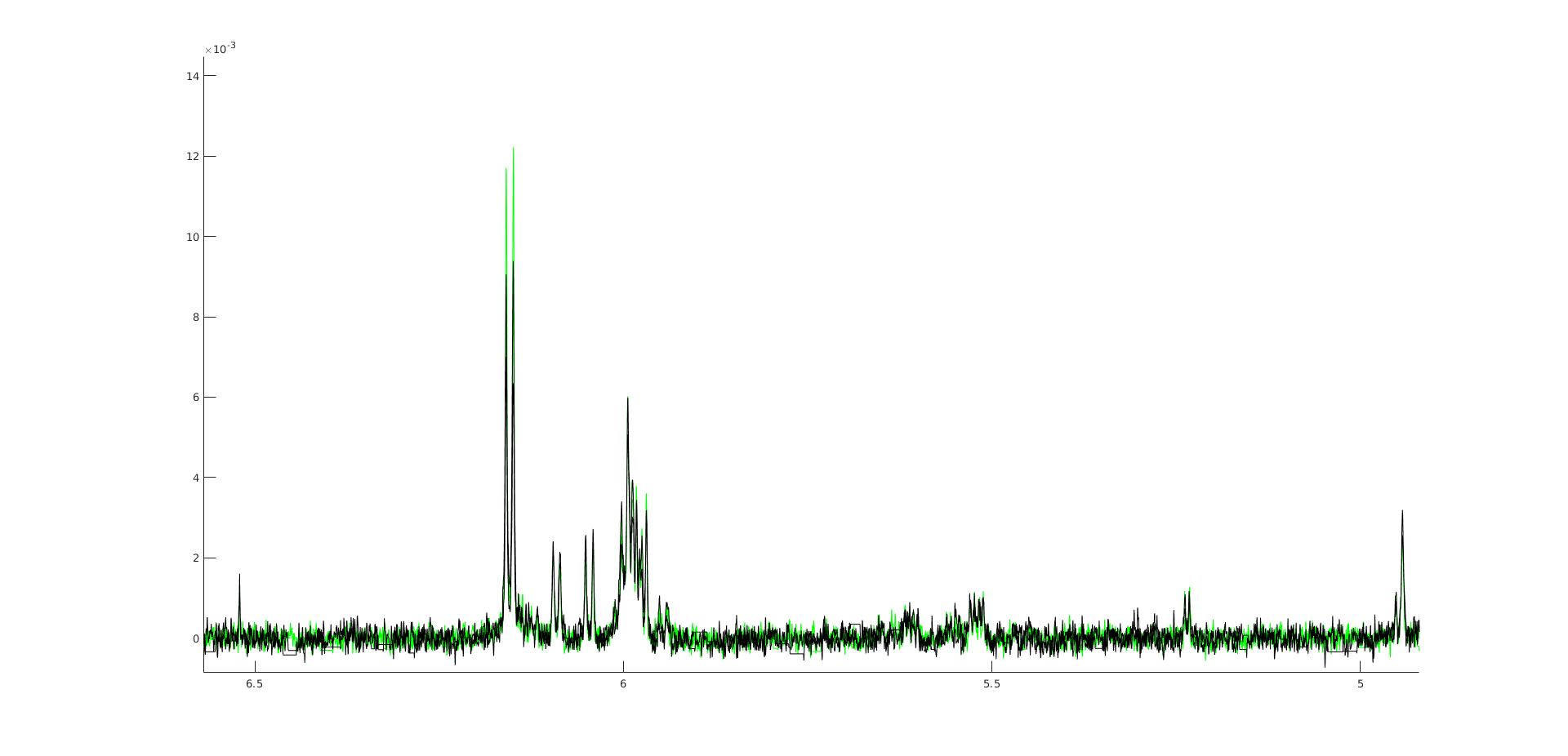


**A**


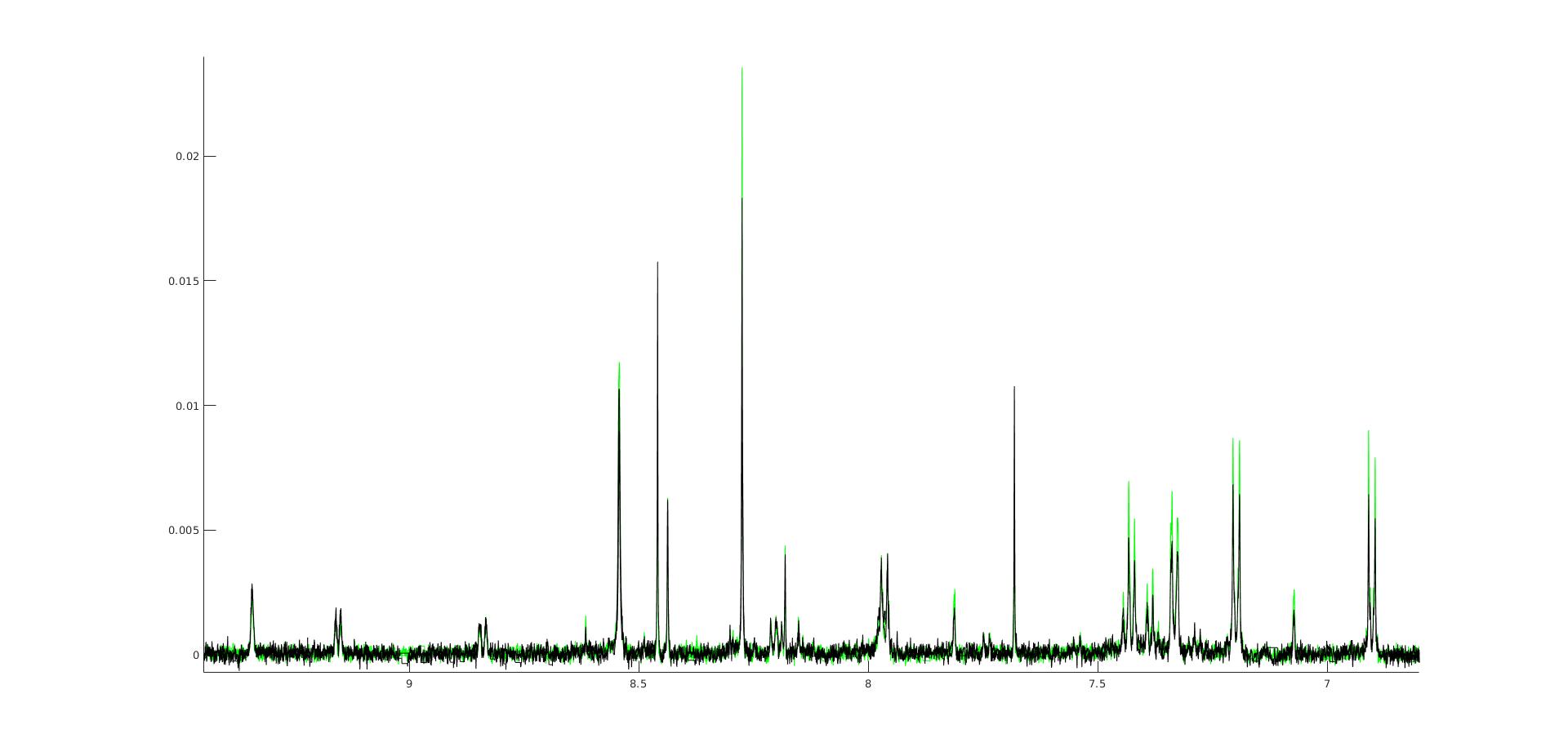


**B**

**Supplementary Figure S5.** Zoomed in part of the ^1^H NMR spectra representing D283_OTX versus Ctrl overlay with D283_OTX in black and D283_Ctrl in green. The panels A and B depict the following zoomed in regions: 5-7 ppm and 7-9 ppm, respectively.


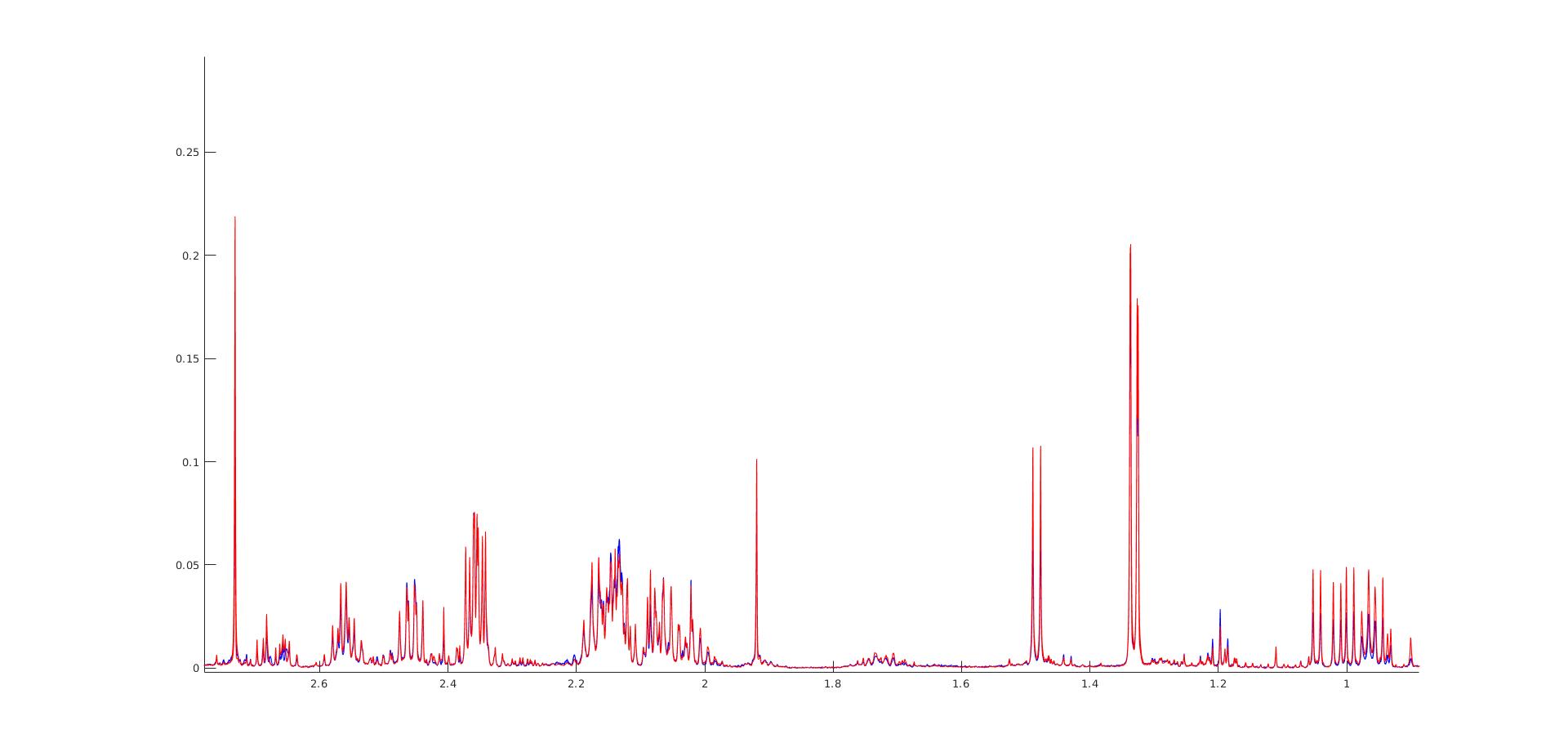


**A**


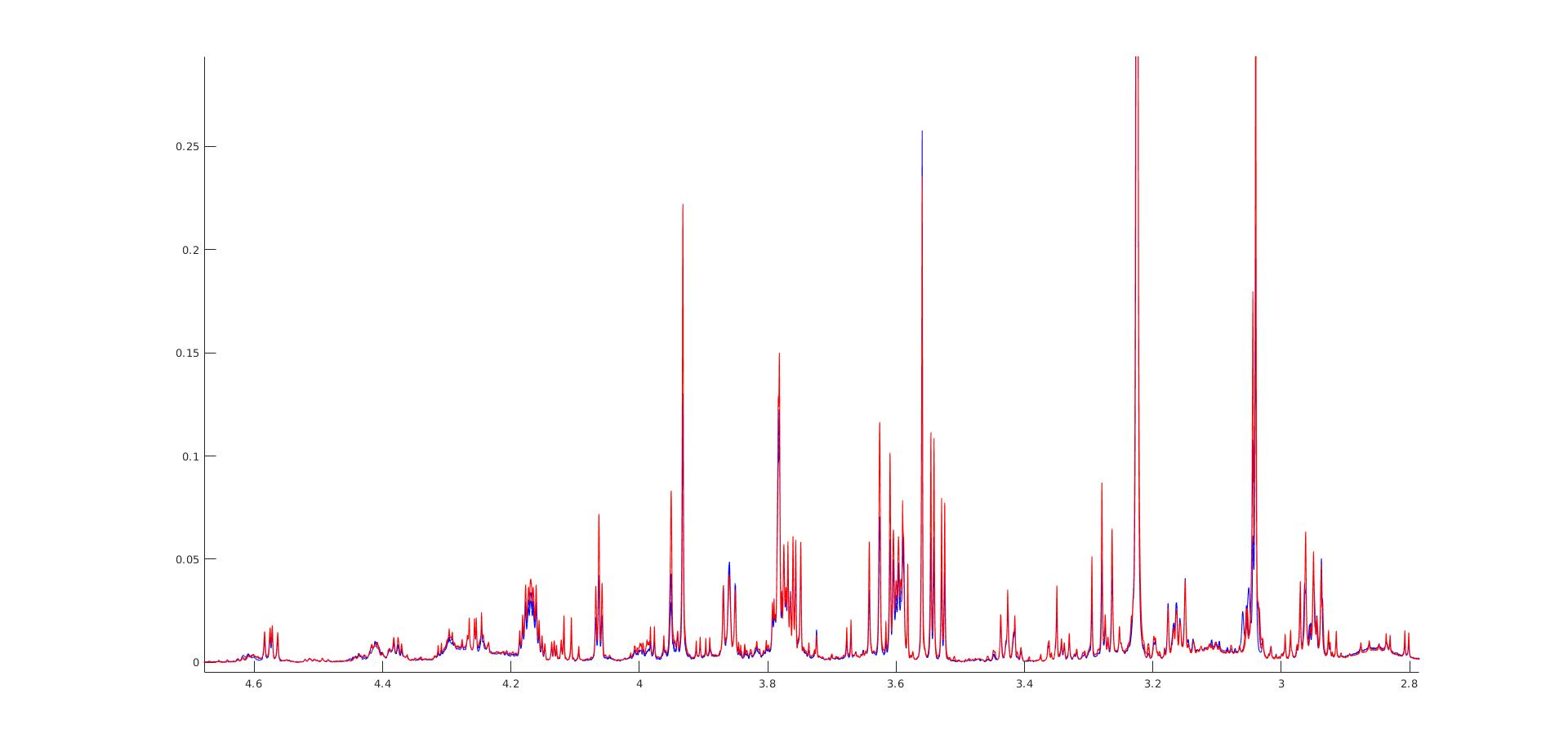


**B**

**Supplementary Figure S6.** Zoomed in part of the ^1^H NMR spectra representing D458_OTX versus Ctrl overlay with D458_OTX in red and D458_Ctrl in blue. The panels A and B depict the following zoomed in regions: 1-3 ppm and 3-5 ppm, respectively.


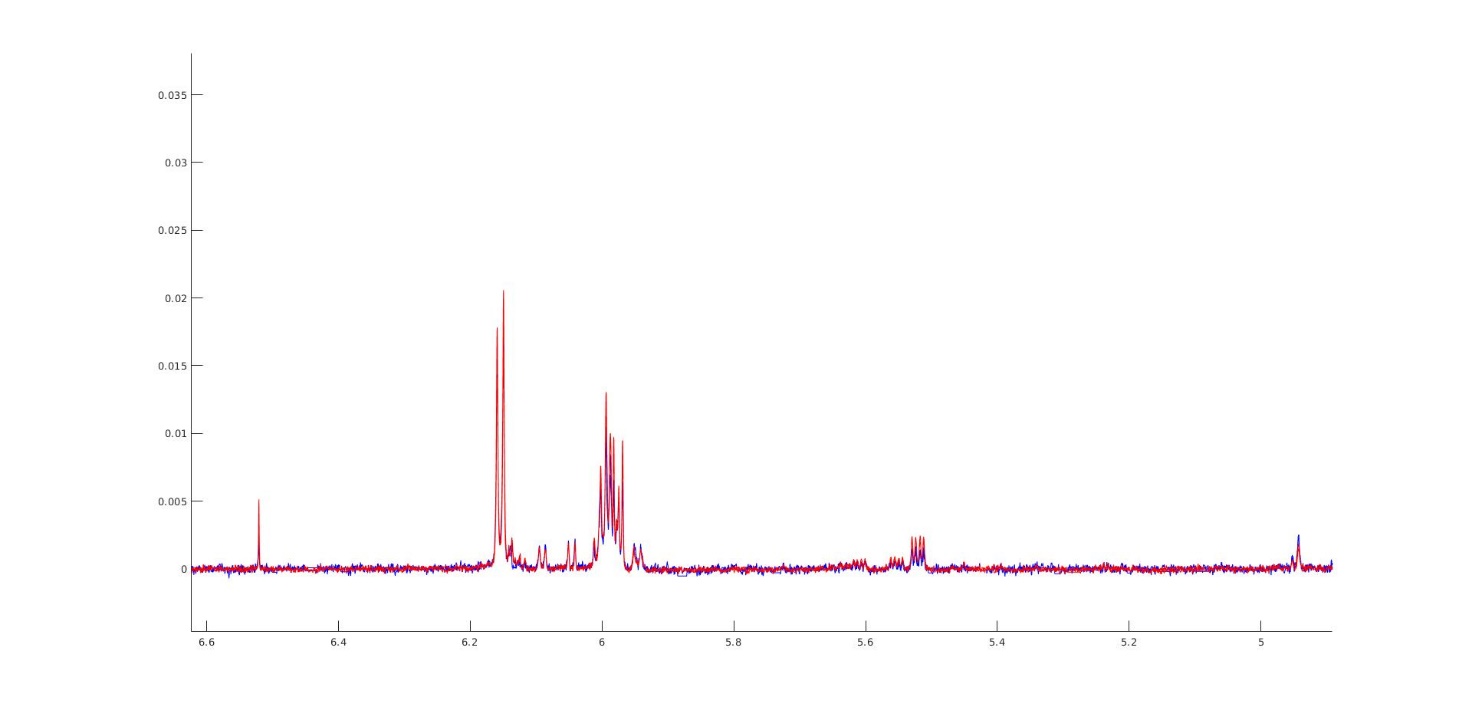


**A**


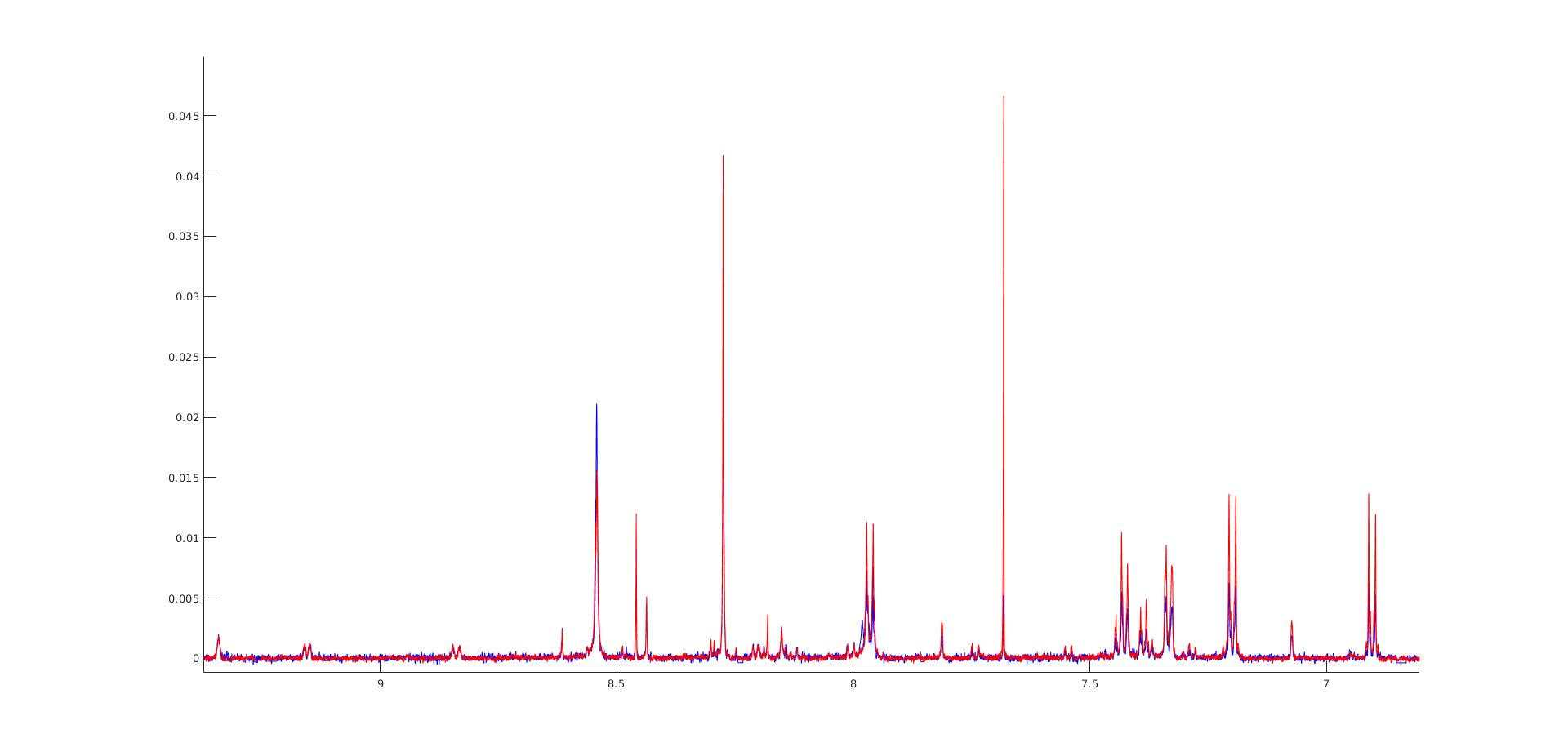


**B**

**Supplementary Figure S7.** Zoomed in part of the ^1^H NMR spectra representing D458_OTX versus Ctrl overlay with D458_OTX in red and D458_Ctrl in blue. The panels A and B depict the following zoomed in regions: 5-7 ppm and 7-9 ppm, respectively.

^1^H


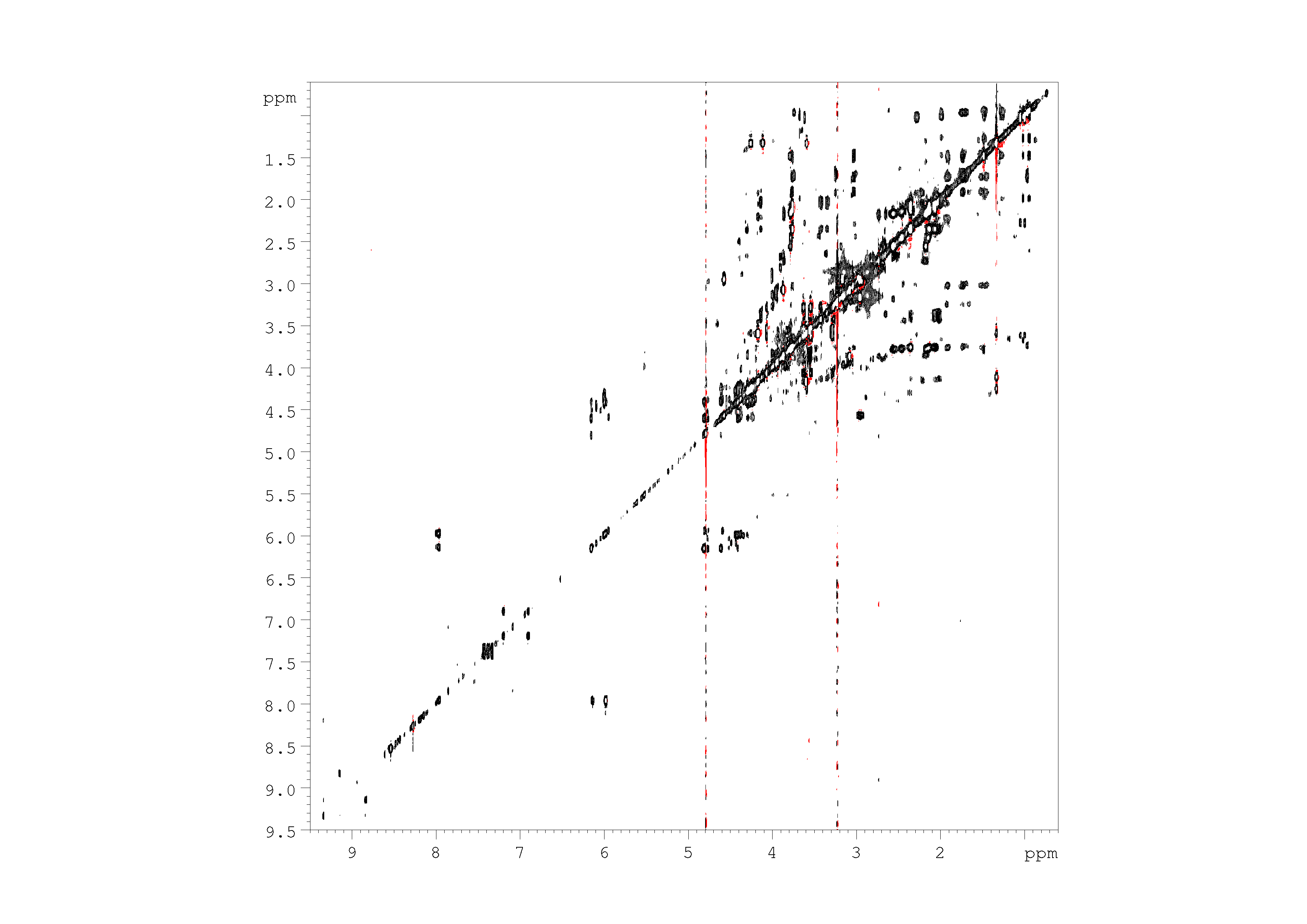


**EtOH**

**Hist**

**PhoCho**

**GSH**

**Val**

**Ile**

**Leu**

**Lac**

**Thr**

**Ala**

**Glu**

**Gln**

**NAD+**

**GlyCho**

**Phe**

**Tyr**

**CTP**

**Fum**

**Pro**

**Myo**

**Hyptau**

**Cit**

**Ace**

**Tau**

**Putr**

**Lys**

**ATP**

**N-Asp**

**UDP**

**Asp**

**UDP-Sugars 1.2 and 3**

**Gluc**

^1^H

^1^H

^1^H

**Supplementary Figure S8.** Plot of [^1^H-^1^H] TOCSY NMR spectrum showing key correlations between the identified metabolites that aided metabolite identification. Spectra were recorded on a Bruker Avance NEO 600 MHz NMR spectrometer equipped with a TCI CryoProbe Prodigy with the pulse sequence “dipsi2gpphzs”, slightly modified to include presaturation, at 298K, with eight scans, 256 t1 increments, a spectral width of 13.7 ppm in both dimensions and a relaxation delay of 2s.


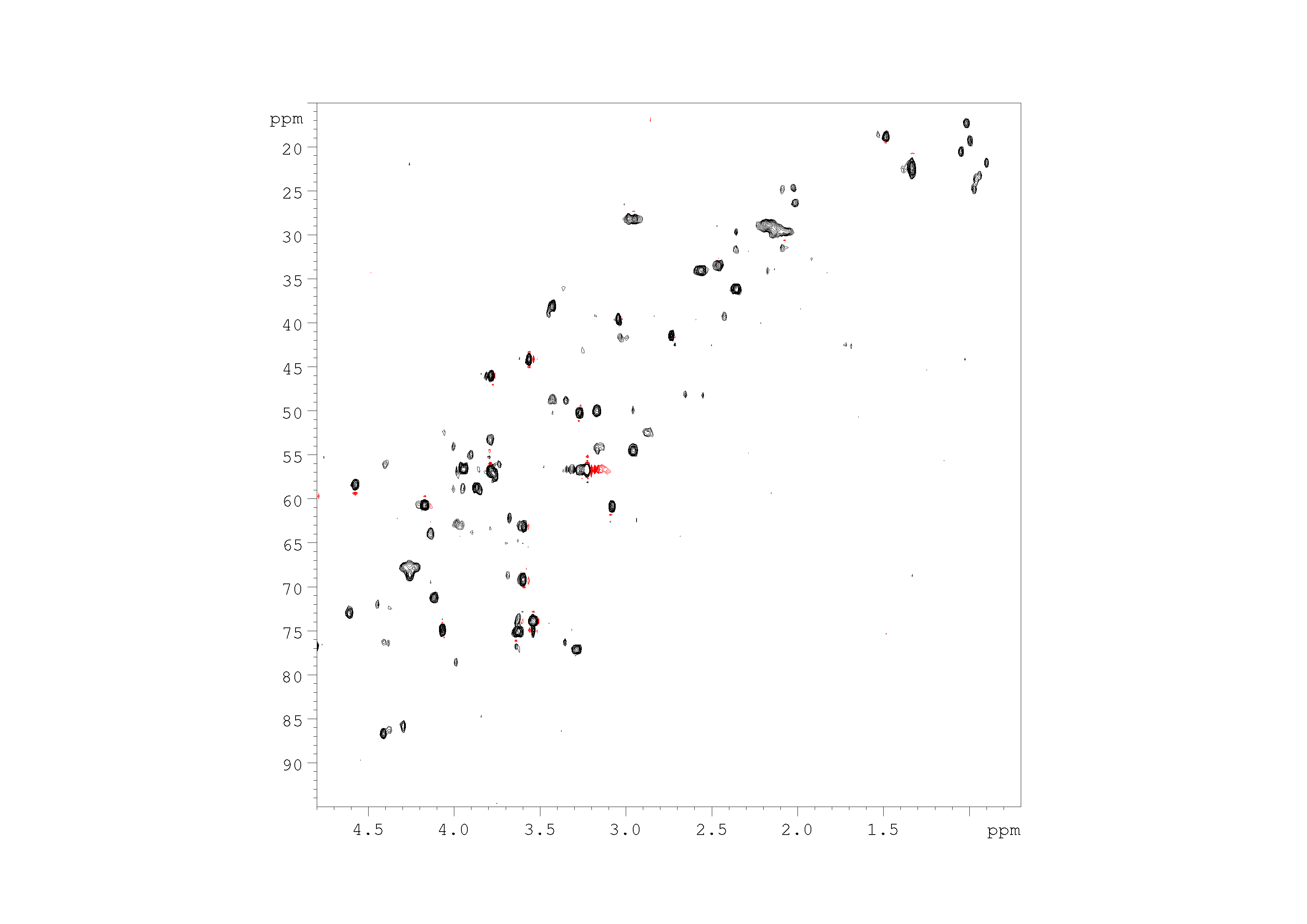


**Pan**

**Leu**

**Val**

**Ile**

**Myo**

**Scyl**

**Lac**

**Ala**

**GSH**

**PhosCho**

**Pyr**

**Suc**

**Cit**

**N-Asp**

**Thr**

**Glu**

**Gln**

**GSH**

**Putr**

**Lys**

**Ser**

**Gly**

**Hyp**

**Tau**

**Crea**

^1^H

^13^C

**Supplementary Figure S9.** Aliphatic region plot of [^1^H-^13^C] HSQC NMR spectrum showing unique cross-peaks from metabolites that aided identification. Spectra were recorded on a Bruker Avance NEO 600 MHz NMR spectrometer equipped with a TCI CryoProbe Prodigy with the pulse sequence “hsqcetgpsisp”, at 298K, with 16 scans, 256 t_1_ increments, a spectral width of 170 ppm in the ^13^C dimension and 12 ppm in the ^1^H dimension, and a relaxation time of 2s.


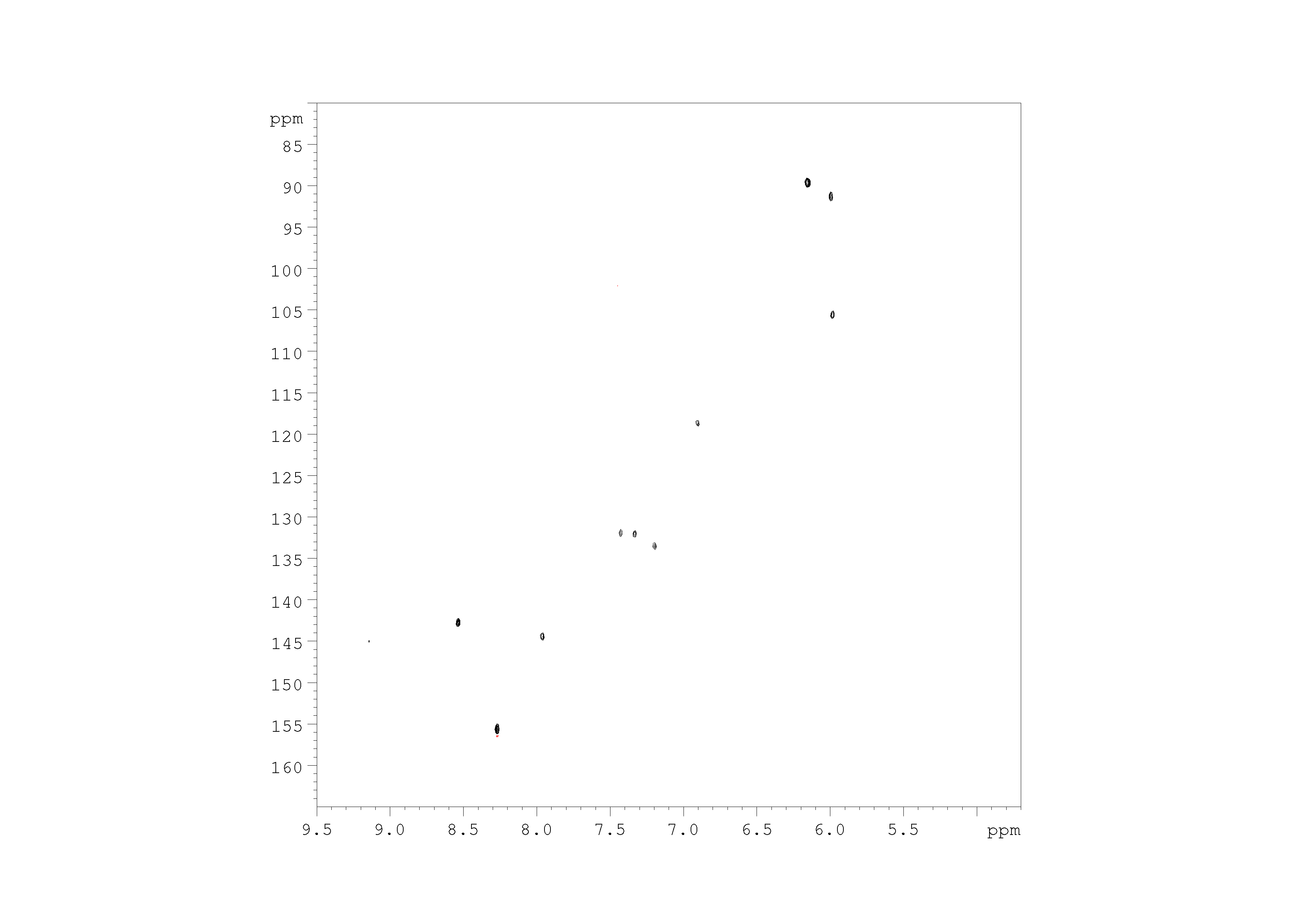


**Tyr**

**Phe**

**For**

**ATP**

**CTP**

^13^C

^1^H

**Supplementary Figure S10.** Aromatic region plot of [^1^H-^13^C] HSQC NMR spectrum showing key correlations between the identified metabolites that aided metabolite identification. Spectra were recorded a Bruker Avance NEO 600 MHz NMR spectrometer equipped with a TCI CryoProbe Prodigy with the pulse sequence “hsqcetgpsisp”, at 298K, with 16 scans, 256 t_1_ increments, a spectral width of 170 ppm in the ^13^C dimension and 12 ppm in the ^1^H dimension, and a relaxation time of 2s.


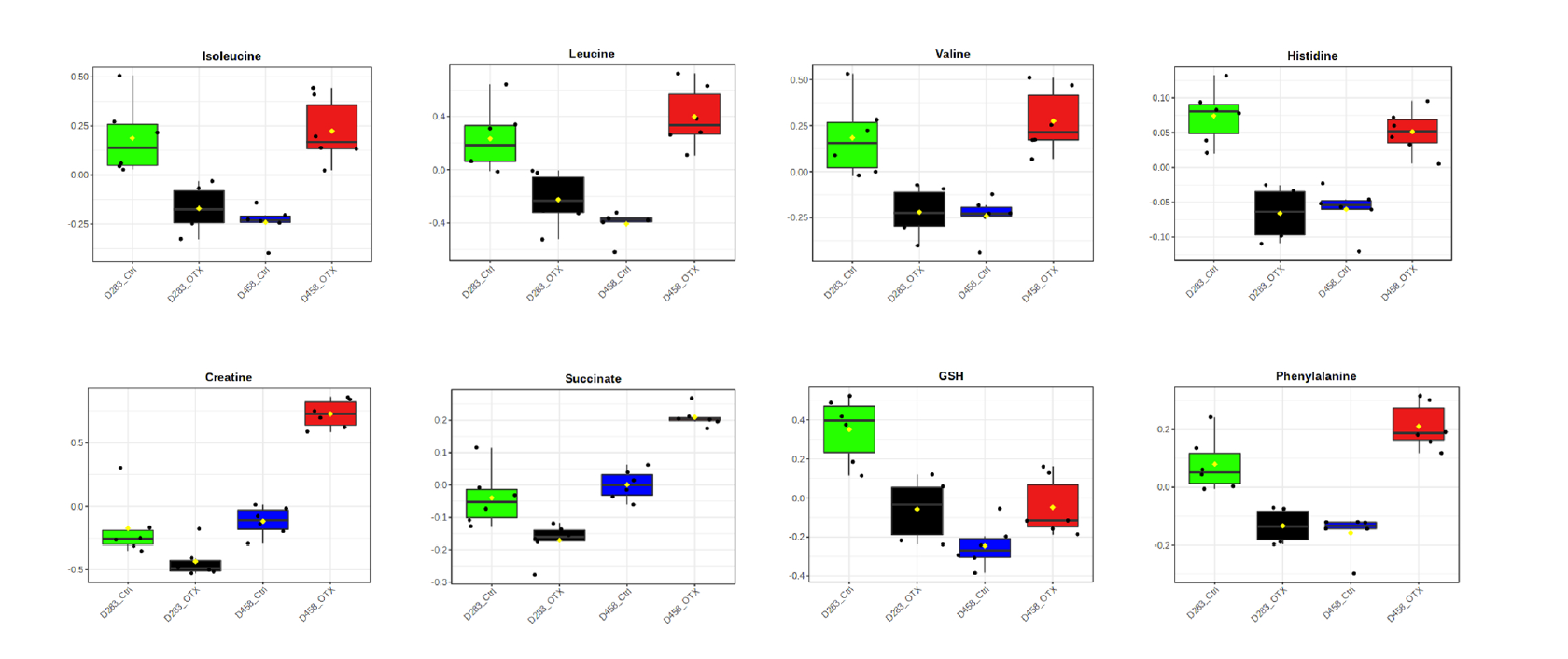


**Supplementary Figure S11.** Boxes depicting metabolites that are statistically significantly regulated in both D283_OTX *versus* D283_Ctrl and D458_OTX *versus* D458_Ctrl, albeit the changes are different in the two cell lines. D283_Ctrl, D283_OTX, D458_Ctrl, D458_OTX are indicated in green, black, blue and red, respectively.

| **Metabolite** | **Left limit (ppm)** | **Right limit (ppm)** | **Mean value D458_Ctrl (u.a.)** | **S.D. D458_Ctrl (u.a.)** | **Mean value D458_OTX (u.a.)** | **S.D. D458_OTX (u.a.)** | **Mean value D283_Ctrl (u.a.)** | **S.D. D283_Ctrl (u.a.)** | **Mean value D283_OTX (u.a.)** | **S.D. D283_OTX (u.a.)** |
| --- | --- | --- | --- | --- | --- | --- | --- | --- | --- | --- |
| Pantothenate | 0.890 | 0.907 | 0.051283 | 0.015160152 | 0.099864 | 0.011812 | 0.21643 | 0.034363 | 0.245454 | 0.031693 |
| Leucine | 0.950 | 0.983 | 0.510408 | 0.040948534 | 0.821735 | 0.090337 | 0.21643 | 0.095255 | 0.580876 | 0.077063 |
| Valine | 0.984 | 1.005 | 0.277516 | 0.029876695 | 0.421541 | 0.049669 | 0.21643 | 0.058198 | 0.283489 | 0.036214 |
| Isoleucine | 1.006 | 1.026 | 0.240304 | 0.021331727 | 0.357291 | 0.042047 | 0.21643 | 0.046577 | 0.258094 | 0.028859 |
| Alanine | 1.469 | 1.497 | 0.702709 | 0.039483474 | 0.973143 | 0.080887 | 0.21643 | 0.099621 | 0.938538 | 0.104581 |
| Lactate | 1.320 | 1.325 | 0.57261 | 0.084249466 | 0.627656 | 0.145645 | 0.21643 | 0.122634 | 0.468431 | 0.107176 |
| Threonine | 1.325 | 1.330 | 0.603519 | 0.0582287 | 0.638566 | 0.092634 | 0.21643 | 0.116473 | 0.625073 | 0.098416 |
| Acetate | 1.916 | 1.924 | 0.388423 | 0.066991485 | 0.334074 | 0.050038 | 0.21643 | 0.05231 | 0.390546 | 0.078698 |
| Glutamate | 2.333 | 2.379 | 2.17862 | 0.262489163 | 2.060269 | 0.250623 | 0.21643 | 0.382026 | 1.366733 | 0.228456 |
| Pyruvate | 2.379 | 2.383 | 0.052525 | 0.005296903 | 0.06061 | 0.00355 | 0.21643 | 0.009707 | 0.041406 | 0.006231 |
| Succinate | 2.403 | 2.409 | 0.081433 | 0.006878188 | 0.112767 | 0.004661 | 0.21643 | 0.013188 | 0.055689 | 0.008585 |
| Glutamine | 2.436 | 2.480 | 0.928268 | 0.079940374 | 0.801889 | 0.069192 | 0.21643 | 0.353003 | 0.893629 | 0.171366 |
| N-acetylasparatate | 2.020 | 2.025 | 0.195601 | 0.013918124 | 0.177994 | 0.008741 | 0.21643 | 0.040029 | 0.344391 | 0.037352 |
| Glycine | 3.555 | 3.563 | 0.830441 | 0.093899711 | 0.698948 | 0.031749 | 0.21643 | 0.165865 | 1.138366 | 0.180956 |
| Myo-inositol | 4.054 | 4.073 | 0.44222 | 0.038679525 | 0.704209 | 0.037337 | 0.21643 | 0.042937 | 0.41176 | 0.0605 |
| Phosphocholine | 4.144 | 4.191 | 1.129731 | 0.087077287 | 1.410035 | 0.110949 | 0.21643 | 0.117371 | 1.46251 | 0.130055 |
| Serine | 3.971 | 3.986 | 0.134372 | 0.013180428 | 0.208795 | 0.012368 | 0.21643 | 0.039214 | 0.277833 | 0.040488 |
| Taurine | 3.408 | 3.443 | 0.40489 | 0.066977572 | 0.495702 | 0.053157 | 0.21643 | 0.18083 | 1.281446 | 0.182177 |
| GSH | 4.558 | 4.588 | 0.273743 | 0.029744937 | 0.325645 | 0.039533 | 0.21643 | 0.043225 | 0.323252 | 0.039364 |
| Creatine | 3.926 | 3.937 | 0.558904 | 0.054092896 | 0.955392 | 0.053381 | 0.21643 | 0.113056 | 0.409864 | 0.06274 |
| Phenylalanine | 7.410 | 7.455 | 0.098879 | 0.012065295 | 0.162513 | 0.013855 | 0.21643 | 0.016234 | 0.10309 | 0.009775 |
| Tyrosine | 6.883 | 6.919 | 0.071958 | 0.00544513 | 0.143943 | 0.011494 | 0.21643 | 0.011793 | 0.088262 | 0.010431 |
| Fumarate | 6.511 | 6.534 | 0.006472 | 0.003341091 | 0.011899 | 0.004934 | 0.21643 | 0.003843 | 0.003691 | 0.001601 |
| CTP | 7.943 | 7.981 | 0.129178 | 0.026838187 | 0.190755 | 0.014288 | 0.21643 | 0.026148 | 0.098639 | 0.02594 |
| UDP-N-acetylglucosammine | 5.503 | 5.536 | 0.031035 | 0.007245042 | 0.056338 | 0.004019 | 0.21643 | 0.005731 | 0.024264 | 0.003909 |
| NAD+ | 9.334 | 9.349 | 0.018581 | 0.001756285 | 0.017528 | 0.00077 | 0.21643 | 0.002577 | 0.032384 | 0.001863 |
| Ethanol | 1.181 | 1.212 | 0.267083 | 0.04640508 | 0.208307 | 0.037025 | 0.21643 | 0.02385 | 0.246352 | 0.062845 |
| Glycerophosphocholine | 3.231 | 3.238 | 0.179963 | 0.023410409 | 0.250153 | 0.023785 | 0.21643 | 0.023957 | 0.32446 | 0.02741 |
| Histidine | 7.061 | 7.086 | 0.016636 | 0.00237383 | 0.024723 | 0.002283 | 0.21643 | 0.002898 | 0.016205 | 0.002711 |
| Putrescine | 1.754 | 1.801 | 0.181906 | 0.04009436 | 0.168072 | 0.036798 | 0.21643 | 0.07178 | 0.222575 | 0.046073 |
| Aspartate | 2.791 | 2.813 | 0.218654 | 0.014690615 | 0.243686 | 0.009237 | 0.21643 | 0.014978 | 0.233414 | 0.033717 |
| Glucose | 5.226 | 5.246 | 0.012948 | 0.011337783 | 0.013593 | 0.0096 | 0.21643 | 0.007159 | 0.018458 | 0.020343 |
| UDP-sugar | 5.539 | 5.569 | 0.021898 | 0.003380106 | 0.034626 | 0.006357 | 0.21643 | 0.002253 | 0.012747 | 0.005537 |
| UDP-glucose | 5.594 | 5.622 | 0.024275 | 0.006513317 | 0.026174 | 0.004166 | 0.21643 | 0.003194 | 0.017934 | 0.00471 |
| ATP | 6.144 | 6.171 | 0.470085 | 0.034546203 | 0.463328 | 0.048379 | 0.21643 | 0.073179 | 0.368866 | 0.054477 |
| Formate | 8.452 | 8.465 | 0.051795 | 0.008435173 | 0.045627 | 0.008661 | 0.21643 | 0.007661 | 0.054097 | 0.009407 |
| Proline | 4.134 | 4.141 | 0.076857 | 0.008430818 | 0.084534 | 0.013207 | 0.21643 | 0.015224 | 0.099627 | 0.016326 |
| Scyllo-inositol | 3.327 | 3.332 | 0.106602 | 0.003969647 | 0.114962 | 0.008634 | 0.21643 | 0.010761 | 0.134415 | 0.014368 |
| Hypotaurine | 3.370 | 3.380 | 0.034821 | 0.003549597 | 0.034557 | 0.002188 | 0.21643 | 0.005851 | 0.062663 | 0.006458 |
| Citric acid | 2.643 | 2.650 | 0.049701 | 0.004352641 | 0.054139 | 0.003477 | 0.21643 | 0.009024 | 0.085198 | 0.007321 |
| UDP | 5.930 | 5.958 | 0.070524 | 0.008696588 | 0.075744 | 0.004367 | 0.21643 | 0.017534 | 0.060707 | 0.010229 |

**Supplementary Table S1.** ^1^H NMR Chemical shift values, Means and Standard Deviations (S.D.) of the identified metabolites in the cellular extract.
